# Single-cell multiplex chromatin and RNA interactions in ageing human brain

**DOI:** 10.1038/s41586-024-07239-w

**Published:** 2024-03-27

**Authors:** Xingzhao Wen, Zhifei Luo, Wenxin Zhao, Riccardo Calandrelli, Tri C. Nguyen, Xueyi Wan, John Lalith Charles Richard, Sheng Zhong

**Affiliations:** 1 Program in Bioinformatics and Systems Biology, University of California San Diego, La Jolla, CA USA; 2 Shu Chien-Gene Lay Department of Bioengineering, University of California San Diego, La Jolla, CA USA; 3 Institute of Engineering in Medicine, University of California San Diego, La Jolla, CA USA; 4 Present Address: Department of Genetics, School of Medicine, Stanford, CA, USA

**Keywords:** Biological techniques, Genomics, Computational biology and bioinformatics

## Abstract

Dynamically organized chromatin complexes often involve multiplex chromatin interactions and sometimes chromatin-associated RNA^1–3^. Chromatin complex compositions change during cellular differentiation and ageing, and are expected to be highly heterogeneous among terminally differentiated single cells^4–7^. Here we introduce the multinucleic acid interaction mapping in single cells (MUSIC) technique for concurrent profiling of multiplex chromatin interactions, gene expression and RNA–chromatin associations within individual nuclei. When applied to 14 human frontal cortex samples from older donors, MUSIC delineated diverse cortical cell types and states. We observed that nuclei exhibiting fewer short-range chromatin interactions were correlated with both an ‘older’ transcriptomic signature and Alzheimer’s disease pathology. Furthermore, the cell type exhibiting chromatin contacts between *cis* expression quantitative trait loci and a promoter tends to be that in which these *cis* expression quantitative trait loci specifically affect the expression of their target gene. In addition, female cortical cells exhibit highly heterogeneous interactions between XIST non-coding RNA and chromosome X, along with diverse spatial organizations of the X chromosomes. MUSIC presents a potent tool for exploration of chromatin architecture and transcription at cellular resolution in complex tissues.

## Abstract (web summary)

We introduce multinucleic acid interaction mapping in single cells (MUSIC), for concurrent profiling of multiplex chromatin interactions, gene expression and RNA–chromatin associations within individual nuclei, as a tool for exploring chromatin architecture and transcription.

## Main

Three-dimensional folding of the genome is known to exhibit dynamic changes during cellular differentiation processes and demonstrates heterogeneity among terminally differentiated single cells^4–7^. Although its regulatory role in the expression of specific genes is well established^8–10^, the extent to which the three-dimensional genome structure impacts the expression of most genes remains a topic of debate^11^. Given the pronounced heterogeneity observed in chromatin structure and gene expression across individual cells^12–14^, a comprehensive understanding of the relationship between three-dimensional genome structure and gene expression at single-cell resolution is necessary. Therefore, the development of single-cell multimodal technologies capable of simultaneous profiling of chromatin conformation and gene expression is instrumental for elucidation of these intricate relationships.

Single-cell multiomic technologies enable joint analysis of chromatin conformation and gene expression^15–19^ (Supplementary Table 1 and Supplementary Note 1). Despite these technical advances, the simultaneous profiling of multiplex chromatin interactions (co-complexed DNA sequences), gene expression and RNA–chromatin associations from a single cell remains challenging. To fill this gap we developed the technique multinucleic acid interaction mapping in single cells (MUSIC), which enables the simultaneous profiling of gene expression and co-complexed DNA sequences with or without co-complexed RNA at the single-cell level.

The architecture of chromatin can encompass both pairwise and multiplex chromatin interactions, highlighting the intricate nature of chromatin complexes^12,20–22^. ChIA-Drop has facilitated the mapping of multiplex chromatin interactions at single-complex resolution from bulk cells, showing that multiplex chromatin interactions are prevalent in *Drosophila*^20^. The MUSIC technique expands the capability of evaluating the composition of pairwise and multiplex chromatin interactions in individual human cells at single-cell resolution.

In addition to DNA, chromatin complexes can also encompass RNA molecules, introducing another layer of complexity to chromatin architecture^1,2,23^. Chromatin-associated RNA has been shown to contribute to the regulation of gene expression^1,24,25^. For instance, the accumulation of XIST long non-coding RNA (lncRNA) on the X chromosome (XIST–chromosome X association) is crucial for the silencing of one of the two X chromosomes in female cells, a process known as X chromosome inactivation. Various human tissues exhibit both shared and tissue-specific incomplete X chromosome inactivation genes (that is, genes that escape from inactivation) that are expressed from the silenced X chromosome^26^. Genes with incomplete X chromosome inactivation can show higher expression levels in women, potentially contributing to sex differences in disease susceptibility^27^. Recent advancements have enabled genome-wide mapping of RNA–chromatin associations in bulk cells^1,28–32^. With the application of MUSIC, we can now obtain RNA–chromatin association maps at the single-cell level. Using MUSIC, we uncover cellular heterogeneity in XIST–chromosome X association levels (XAL) within the female cortex and explore the covariation between XAL and chromatin interactions among female cortical cells.

## Design and workflow

**Fig. 1:**
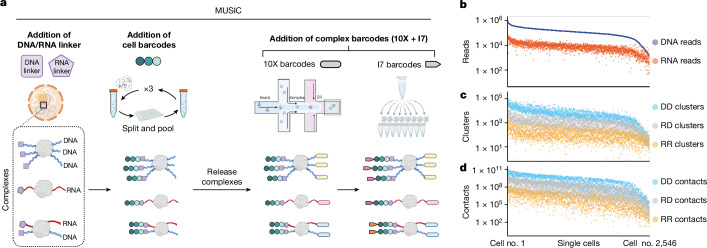
MUSIC workflow and statistics. **a**, Schematic view of MUSIC experimental pipeline. **b**–**d**, Summary of MUSIC data in H1 cells. Numbers of uniquely mapped non-duplicate reads (**b**), clusters (**c**) and pairwise contacts (**d**) in every H1 cell (column, *n* = 2,546). DNA–DNA (DD, blue), RNA–DNA (RD, grey) and RNA–RNA (RR, yellow) clusters are counted separately. Multiplex interactions are projected to pairwise interactions, with the numbers of pairwise contacts shown in **d**.

Development of MUSIC technology is guided by three specific design goals which, collectively, enable the joint profiling of gene expression, co-complexed DNA sequences and RNA–chromatin associations from the same nucleus (Fig. 1a). The first goal is to construct RNA and fragmented DNA into a single sequencing library and identify which RNA and DNA sequences originated from the same nucleus. This goal is achieved by labelling all RNA and fragmented DNA in the same nucleus with a unique cell barcode, which enables the identification and matching of RNA and DNA sequences originating from the same nuclei.

The second goal is to distinguish RNA inserts from DNA inserts in the sequencing library. To achieve this, distinct nucleotide sequences are used for the RNA and DNA linkers, which are ligated to the RNA and DNA molecules, respectively. These linkers are sequenced alongside the RNA and DNA inserts, enabling the differentiation of RNA and DNA molecules within the sequencing data. The third goal is to capture and identify DNA–DNA and RNA–DNA associations, including multiway contacts. To achieve this, each molecular complex is labelled with a unique complex barcode. A molecular complex can encompass various combinations of DNA and RNA, including (1) an isolated RNA molecule, (2) an isolated DNA fragment, (3) multiple DNA fragments, (4) multiple RNA molecules, and (5) at least one RNA molecule and at least one DNA fragment. These complex barcodes, together with the cell barcodes, allow for the identification of co-complexed DNA and/or RNA in each cell.

**Extended Data Fig. 1:**
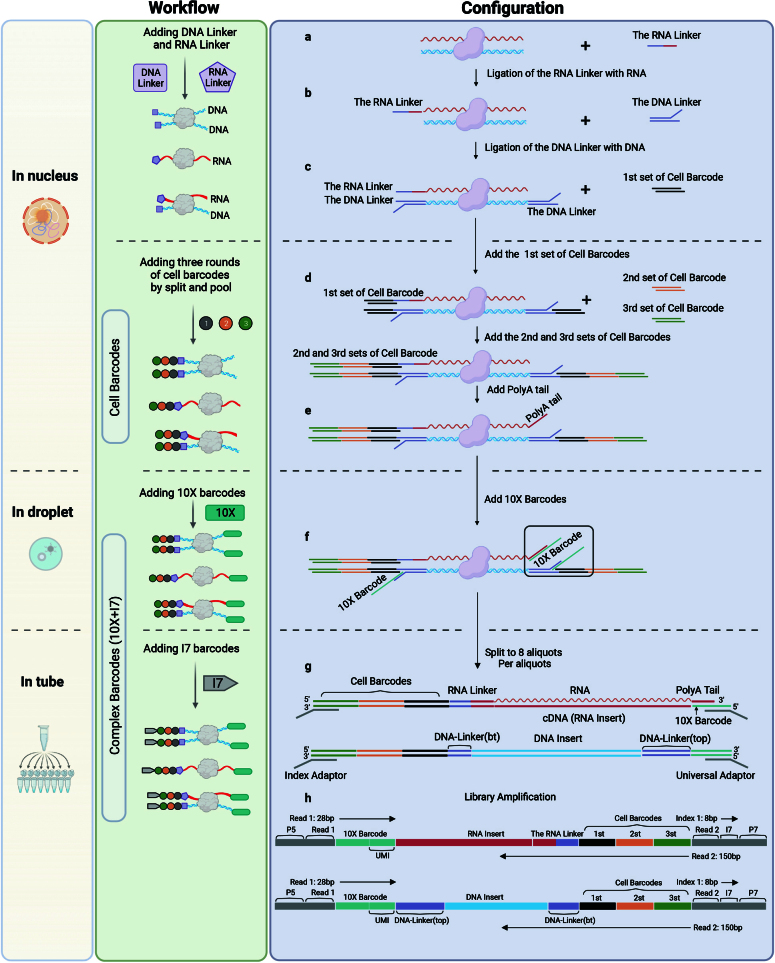
Overview of the MUSIC method. (a) An example chromatin complex with one associated DNA fragment (blue) and one RNA molecule (red) is shown to illustrate the procedure. Other chromatin complexes with any number of associated DNA fragments or RNA molecules are expected to react accordingly. The RNA Linker with half single strand RNA (ssRNA, red) and half single strand DNA (ssDNA, purple). (b) The ligation product with a RNA linker conjugates with the Y-shaped DNA Linker that contains a double strand region (dsDNA) and single strand regions (top-ssDNA and bottom-ssDNA). (c) The ligation product with both DNA and RNA linkers. (d-e) Addition of the three sets of Cell Barcodes (color coded in black, yellow, green) and A-tail. (f) Reaction in a 10X GEM system. (g) Aliquoting the output of the 10X GEM system for library preparation. (h) The sequence configuration of the constructed sequencing library. For Illumina paired-end sequencing, Read1 covers the 28 bp 10X Barcode that consists of a 16 bp 10X GEM Barcode and 12 bp 10X UMI. Index 1 covers the 8 bp I7 Barcode, and Read2 covers the Cell Barcodes as well as the RNA Linker and the RNA insert or the DNA Linker and the DNA insert.

The MUSIC workflow contains two major steps (Supplementary Note 2). The first of these ligates the RNA linker to the RNA molecules and the DNA linker to the fragmented DNA and adds the cell barcodes (Extended Data Fig. 1a–e). The second step adds a complex barcode to any RNA or DNA within the same molecular complex. The complex barcode consists of a 10X barcode and an I7 barcode (Extended Data Fig. 1f,g). The final sequencing library is sequenced with a 28 base pair (bp) read1 sequence, an 8 bp index sequence that is the I7 barcode and a 150 bp read2 sequence (Extended Data Fig. 1h). The 28 bp read1 corresponds to the 10X barcode, which consists of a 16 bp 10X GEM barcode and a 12 bp 10X unique molecular identifier (UMI). Read2 contains the third, second and first cell barcodes, the RNA or DNA linker sequence and the RNA or DNA insert. It should be noted that each read pair is designed to capture only one insert, either an RNA or a DNA insert, because read1 is dedicated to reading the 10X barcode. This design differs from several ligation-based methods such as Hi-C^33^ and iMARGI^28,29^ in which each read pair represents two inserts.

## RNA–chromatin interactions in embryonic stem cells

We applied MUSIC to analyse a mixed population of H1 human and E14 mouse embryonic stem cells. The resulting mixed-species MUSIC library was sequenced on a NovaSeq platform, generating 3,067,956,666 read pairs. These read pairs resolved 533,233,368 uniquely mapped, non-duplicate and barcode-complete (containing cell barcode, 10X barcode, I7 barcode and either DNA or RNA linker) (UMNDBC) read pairs (Supplementary Table 3). According to the experimental design, because each UMNDBC read pair contains only one DNA or RNA insert we will refer to a UMNDBC read pair as either a DNA or RNA read. This mixed-species dataset showed low species-mixing rates at both the cellular and chromatin complex level, supporting the ability of MUSIC to generate data at both single-cell and single-complex resolution (Extended Data Fig. 3a,b and Supplementary Note 3).

We identified 2,546 human H1 cells from this dataset. Each H1 cell contained an average of 144,049 UMNDBC DNA reads and 11,384 UMNDBC RNA reads, corresponding to 7,036 DNA-only clusters, 232 RNA-only clusters and 1,170 RNA–DNA clusters (Fig. 1b,c) which, based on an established procedure^12^, can be resolved into 2,639,302,084 co-complexed DNA–DNA pairs, 7,089,720 co-complexed RNA–RNA pairs and 250,525,581 co-complexed RNA–DNA pairs (Fig. 1d). In total there were 18,144,410 non-singleton DNA-only clusters accounting for 55,670,578 DNA reads, 324,121 RNA-only clusters accounting for 835,184 RNA reads and 2,401,392 RNA–DNA clusters accounting for 13,151,716 RNA reads and 216,515,595 DNA reads (Extended Data Fig. 3c). Among non-singleton DNA–DNA clusters, 13,111,228 (72.26%) contained two DNA reads corresponding to pairwise interactions and 5,033,182 (27.74%) contained three or more DNA reads responding to multiplex interactions (Extended Data Figs. 3d and 4). Among RNA–DNA clusters 1,009,706 (42.05%) contained two reads—that is, one DNA read and one RNA read, 783,709 (32.64%) contained between three and ten reads and 607,977 (25.32%) contained more than ten reads.

**Fig. 2:**
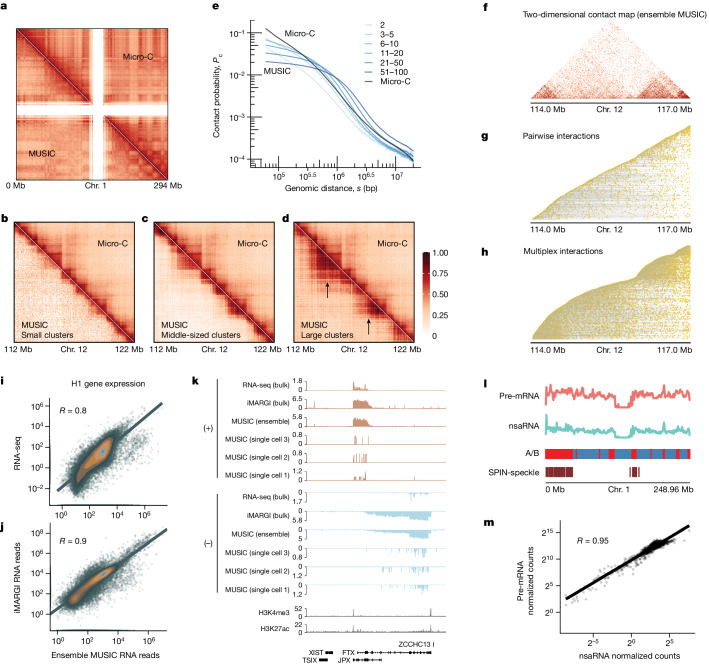
MUSIC data for H1 cells. **a**, Comparison of Micro-C- and ensemble MUSIC-derived chromatin contact maps on chromosome (chr.) 1 at 1 Mb resolution. **b**–**d**, Chromatin contact maps based on the ensemble of small (**b**), middle-sized (**c**) and large (**d**) clusters. Resolution, 50 kb. Arrows represent contacts between nested TADs. **e**, *P*_c_(*s*) curves showing the frequency of chromatin contacts (*P*_c_) versus genomic distance (*s*) for MUSIC DNA-only clusters of varying size and Micro-C. **f**–**h**, Comparison of two-dimensional contact map from ensemble MUSIC data (**f**) with stacked maps of distinct DNA–DNA clusters. Each row represents a cluster, ordered by the smallest genomic coordinate of any DNA read. Yellow dots denote genomic locations of DNA reads within a cluster. **g**, Clusters with two DNA reads (pairwise interactions). **h**, Clusters with three or more DNA reads (multiplex interactions). **i**,**j**, Scatterplots of RNA levels as measured by reads per kilboase (RPK) for every gene (dot) in ensemble MUSIC versus RNA-seq (**i**) and versus iMARGI (**j**). *R* denotes Spearman correlation coefficient. **k**, RNA reads from RNA-seq, iMARGI and MUSIC mapped to both strands (+ and −) in human H1 cells. Ensemble and individual MUSIC data from three single cells are shown. **l**, Distribution of chromatin-associated pre-mRNA and nsaRNA on chromosome 1 as measured by ensemble MUSIC in H1 cells. Micro-C-derived A and B compartments are in red and blue, respectively. Speckle compartmentalization derived from the SPIN model is denoted in the SPIN-speckle track. **m**, Scatter plot of normalized counts of pre-mRNA and nsaRNA reads in every 1 Mb genomic bin (dot) across the entire genome, based on ensemble MUSIC data for H1 cells.

We compared MUSIC ensemble DNA reads with Micro-C data generated from the same human H1 cell line and cultured under the standard operating protocol recommended by the 4D Nucleome Consortium. The contact map of MUSIC DNA–DNA clusters reproduced the structures observed in the Micro-C-derived contact map (4DN data portal: 4DNFI2TK7L2F^34^) (Fig. 2a), resulting in a similar distribution of compartment scores across the genome (Extended Data Fig. 5a). We compared the variously sized MUSIC DNA–DNA clusters (Fig. 2b–d, bottom left), using the same Micro-C dataset as a reference (Fig. 2b–d, top right). At the topologically associating domain (TAD) level, MUSIC small DNA–DNA clusters (between two and ten DNA reads per cluster) primarily contained contacts within TADs (50 kb resolution) (Fig. 2b). MUSIC middle-sized (11–50 reads per cluster) and large clusters (51–100 reads per cluster) recapitulated the TADs and the nested TAD structure (Fig. 2c,d). Large clusters showed more contacts between nested TADs within a larger TAD (Fig. 2d, arrows).

For visualization of clusters we plotted each one in a row, with every DNA read of that cluster aligned to its respective genomic coordinates. We ordered clusters by the genomic coordinates of their leftmost DNA reads and, in this way, we created a stacked map of the clusters (Fig. 2g,h). By comparison of the two-dimensional contact map based on ensemble MUSIC data (Fig. 2f) with the stacked maps, we observed that pairwise interactions (clusters with two DNA reads) alone poorly reflected the TAD structure (Fig. 2g) whereas multiplex interactions (clusters with three or more DNA reads) recapitulated it (Fig. 2h). Analysis involving downsampling of reads suggested that this difference was not due to variation in read numbers between pairwise and multiplex interactions (Supplementary Note 4 and Extended Data Fig. 5b). This analysis corroborates the variation found in the contact maps of different cluster sizes (Fig. 2b–d), suggesting that the TAD chromatin structure predominantly consists of multiplex chromatin interactions. In addition, compared with pairwise contacts, multiplex interactions showed higher contact frequencies at submegabase to several megabase genomic distances, indicating enrichment of long-range chromatin interactions in the multiplex complexes (Fig. 2e and Supplementary Note 5).

We compared MUSIC ensemble RNA reads (RNA ensemble) from the 2,546 H1 cells with RNA measurements obtained from two bulk assays in H1. Using all 60,719 genes defined in GENCODE v.36, we quantified the RNA level of each gene in terms of reads per kilobase. The RNA levels of the MUSIC RNA ensemble correlated with those of bulk RNA sequencing (RNA-seq; ENCSR000COU^35^) (Fig. 2i; rho = 0.8, *P* < 2.2 × 10^−^^16^). Furthermore, iMARGI is a bulk assay of RNA–chromatin interactions in which the collection of RNA reads from iMARGI measures the transcriptome in nuclei^2^. The RNA ensemble of MUSIC also correlated with that of iMARGI RNA reads (Fig. 2j; rho = 0.9, *P* < 2.2 × 10^−^^16^). This indicates that the gene expression levels quantified by MUSIC ensemble RNA reads are consistent with those obtained from bulk RNA assays. Moreover, MUSIC detects various types of RNA species (Extended Data Fig. 5c,d) and is strand specific (Fig. 2k and Extended Data Fig. 5e). In addition, MUSIC recapitulated the known chromatin association patterns of premessenger RNAs (pre-mRNAs) and nuclear speckle-associated RNAs (nsaRNA) (Fig. 2l,m and Supplementary Note 6).

## A MUSIC map of human frontal cortex

We generated a MUSIC dataset on 14 postmortem samples of human frontal cortex from tissue donors aged 59 years and above^36^ (Supplementary Table 4). This dataset, hereafter referred to as MUSIC FC, resolved 9,087 single nuclei, 755,123,054 UMNDBC DNA reads and 29,319,780 UMNDBC RNA reads (hg38) (Extended Data Fig. 6a,e). MUSIC FC resolved comparable numbers of single-nucleus RNA (snRNA) reads and DNA–DNA contacts versus other methods (Supplementary Note 7 and Extended Data Fig. 6b–d).

**Fig. 3:**
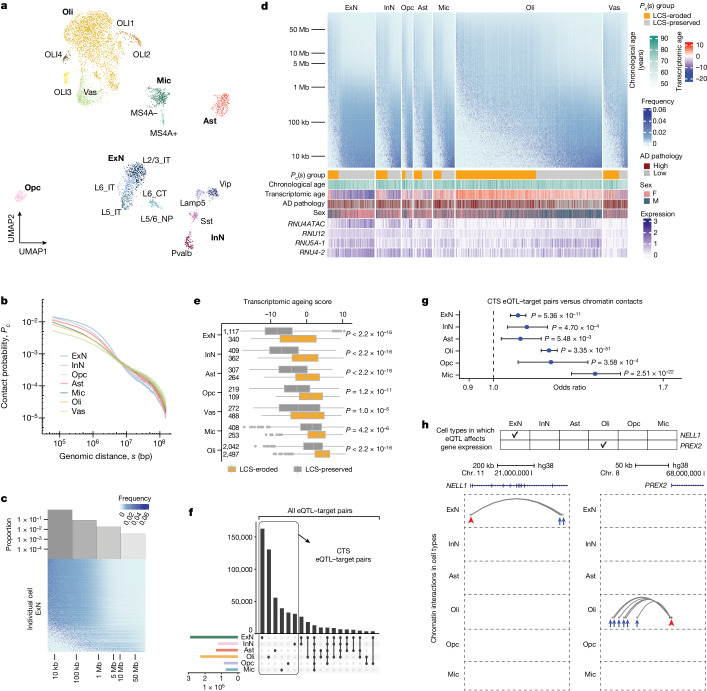
A single-cell map of transcriptome and chromatin conformation in human frontal cortex. **a**, Uniform manifold approximation and projection (UMAP) representation of individual cortical cells based on MUSIC RNA reads. Ast, astrocytes; ExN, excitatory neurons; InN, inhibitory neurons; Oli, oligodendrocytes; Opc, oligodendrocyte precursors; Mic, microglia; Vas, vascular cells. **b**, Chromatin contact frequency (*P*_c_) versus genomic distance (*s*) for each cell type. **c**, Histogram of the proportions of excitatory neurons with their most frequent chromatin interactions in each genomic bin aligned with normalized contact frequency versus genomic distance plot for every excitatory neuron. **d**, Chromatin contact frequency versus genomic distance in individual cortical cells (columns). Rows, genomic bins with exponential size increase. Color scale denotes chromatin contact frequency normalized by bin size. Bottom tracks show *P*_c_(*s*) group, chronological age, transcriptomic age, Alzheimer’s disease (AD) pathology status, sex and expression levels of several genes. **e**, Single-cell transcriptomic age for each cell type coloured by chromatin conformation age. *P* values determined by one-sided Wilcoxon test. Numbers on left indicate sample size. In the box plots, the left, centre and right edges represent the 25th, 50th and 75th quantiles, respectively; whiskers extend to 1.5 times the interquartile range; data points beyond whiskers are outliers. **f**, Upset plot of eQTL–target pairs in every cell type. Box indicates cell-type specific (CTS) eQTL–target pairs. **g**, Association tests between DNA–DNA contacts and cell-type-specific eQTL–target pairs in every cell type. Centre dot denotes odds ratio; whiskers denote 95% confidence interval of odds ratio. *P* values determined by one-sided chi-square test. *n* = 101,785 DNA–DNA contacts. **h**, Examples of cell-type-specific eQTL–target pairs supported by DNA–DNA contacts. Top, tick marks indicate the cell type (column) in which the *cis* eQTLs of a gene affect expression. Bottom, genome track view of supporting DNA–DNA contacts in every cell type (row), which are DNA–DNA contacts (curves) overlapping with *cis* eQTLs (blue arrows) and the promoter of the target gene (red arrowhead).

Clustering analysis based on MUSIC snRNA reads identified seven cell types: excitatory neurons, inhibitory neurons, astrocytes, oligodendrocytes, oligodendrocyte precursors, microglia and vascular cells (Fig. 3a and Supplementary Note 8). The microglial cluster consists of two subclusters, marked by low and high expression levels of *MS4A* genes (Extended Data Fig. 7m–o), which may reflect microglial subpopulations in the chemokine state relative to the interferon state^37^. In addition, a joint analysis of MUSIC FC with a snRNA-seq dataset of human frontal cortex^38^ showed highly consistent clustering structures and clustering-based cell type assignments between the two datasets (Supplementary Note 9).

Stratification analysis, by neither sex (Extended Data Fig. 7c,e) nor individual cortical sample (Extended Data Fig. 7d), substantially affected the proportions of cells in clusters or subclusters, except for a higher number of oligodendrocytes in men compared with women (Extended Data Fig. 7b,c,e). Our data indicate a sex difference in the number of cortical oligodendrocytes in older people (59 years of age or above), which aligns with previous studies showing that the lifespan of oligodendrocytes in female mice is shorter than in male mice^39^. In summary, MUSIC FC data formed clear clusters that correspond with known cortical cell types and cellular states.

## Heterogeneity in chromatin interactions

Bulk analyses of chromatin conformation showed that chromatin interaction frequency (*P*_c_) decreases as genomic distance (*s*) increases, forming an approximately linear relationship on the log–log scale^33^. This trend was also observed in MUSIC FC, in which the aggregate chromatin interaction frequency in the ensemble of single cells decreased with increasing genomic distance (Fig. 3b). Hereafter we refer to this trend as the aggregate *P*_c_–*s* relationship.

At the single-cell level, most single cells also exhibited a reverse correlation between chromatin interactions and genomic distance, whereas a minority of single cells exhibited the highest chromatin interactions and not necessarily at the lowest genomic distances, a deviation from the aggregate *P*_c_–*s* relationship (Fig. 3c,d). This observation is reminiscent of the recently reported increase in ultra-long-range intrachromosomal interactions during ageing in cerebellar granule cells^40^ (Extended Data Fig. 8e). To test whether this observed cellular heterogeneity is compatible with the aggregate *P*_c_–*s* relationship, we binned genomic distances and counted the proportion of single cells showing the highest chromatin interaction in each genomic distance bin (Fig. 3d). The proportion of single cells is lower in the bins of longer genomic distances, conforming to a reverse correlation that is approximately linear in the log–log scale (Fig. 3c). Thus, despite the high degree of cellular heterogeneity, the population summary of single cells reproduces the previously reported aggregate relationship.

Whereas the different cell types exhibited similar aggregate *P*_c_(*s*) curves, these were not identical (Fig. 3b), such differences indicating cell type variations in chromatin conformation. In particular, excitatory neurons exhibited more frequent chromatin interactions within the submegabase range of genomic distances than other cell types (Fig. 3b). Consistent with this aggregate behaviour, excitatory neurons had a larger proportion of single cells exhibiting the most frequent chromatin interactions at the submegabase range compared with other cell types (Fig. 3c and Extended Data Fig. 7f). Together, these observations highlight the influence of cellular composition in each cell type on cell type variation in chromatin conformation.

We compared the cellular heterogeneities in *P*_c_(*s*) and transcriptomic profile. For convenience we will term cells with high chromatin interaction frequencies at low genomic distances as ‘local chromatin structure (LCS)-preserved’ and those with high chromatin interaction frequencies at high genomic distances as ‘LCS-eroded’. We simplified *P*_c_(*s*) for each cell into a singular score—that is, the peak genomic distance of *P*_c_(*s*), which we termed the LCS-erosion score for this cell. A higher LCS-erosion score reflects a greater loss in local chromatin structure. In every cell type we identified those genes with single-cell expression levels that correlated with single-cell LCS-erosion scores. Pathway enrichment analysis showed that these genes are enriched in the expected functions of the corresponding cell type (Extended Data Fig. 8a). For example, in excitatory neurons, LCS-eroded cells exhibited reduced expression of genes associated with axon guidance, ErbB signalling and glutamatergic synapse whereas, in inhibitory neurons, these cells exhibited reduced expression of genes in gamma-aminobutyric acid synthesis. These data suggest that cell-type-specific functions are impaired in LCS-eroded cells.

Next we analysed all cortical cells together and identified those genes with single-cell expression levels that correlated with single-cell LCS-erosion scores. A group of small nuclear RNA genes (*RNU4ATAC*, *RNU4-2*, *RNU5A-1*, *RNU12*) emerged as being highly correlated with LCS-erosion scores, with high expression in LCS-preserved cells in every cell type (Fig. 3d). Small nuclear RNAs are integral components of the spliceosome, and reduced spliceosome fidelity has emerged as a characteristic of cellular senescence and ageing^41,42^. These data, and cell-type-specific analyses, enabled us to speculate that single-cell *P*_c_(*s*) indicates the ‘age’ of that cell. To test this idea, we used the recently published SCALE model^43^ to compute the transcriptomic age of each single cell, which is a model-based weighted average of age-related gene expression in that cell. LCS-eroded cells exhibited higher transcriptomic age than LCS-preserved in every cell type, suggesting that the former are older in transcriptomic age (Fig. 3e). In comparison, the chronological age of a sample (age at death) exhibited only weak correlations with LCS-erosion scores (Extended Data Fig. 8b), suggesting the limited ability of donor chronological age to explain the cellular heterogeneity of LCS-erosion scores (or *P*_c_(*s*)). Together, cells with reduced chromatin contact frequency at smaller genomic distance tended to exhibit older transcriptomic age.

Seven of the fourteen cortex tissues exhibited high pathology of Alzheimer’s disease (Braak score of 4 or above) whereas the other seven exhibited low pathology (Braak score of 3 or less). The single cells of low-pathology samples exhibited lower LCS-erosion scores than those of the high-pathology samples in excitatory neurons, inhibitory neurons, astrocytes, oligodendrocytes and microglia (Extended Data Fig. 8c), showing a correlation between loss of local chromatin structure and high pathology. These data are reminiscent of a recent report on the association of global epigenome dysregulation and Alzheimer’s disease^44^. In addition, regression analysis showed that those factors with the greatest correlations to LCS-erosion score included transcriptomic age, cell type, Alzheimer’s disease pathology and sex (Supplementary Note 10).

## Correlation of eQTL and chromatin contacts

*Cis* expression quantitative trait loci (eQTLs) represent genomic regions in which individual sequence variations contribute to the variation in expression of nearby genes^45^. One recent study reported 481,888 *cis* eQTLs in the human brain^45^. Notably, the majority of these *cis* eQTLs exert their influence on the expression of specific target genes within particular cell types, indicating a strong cell type specificity in eQTL–target pairings (Fig. 3f). The underlying factor responsible for the cell type specificity observed in eQTL–target pairings remains elusive. We compared cell type variation in chromatin contacts (Fig. 3b and Supplementary Fig. 3a) with eQTL–target pairings in an association test. This analysis involved all eQTL–target pairs and all chromatin contacts overlapping with an eQTL and its target gene promoter (supporting DNA–DNA contacts), irrespective of their appearance in the same cell type. This test identified significant enrichment of supporting DNA–DNA contacts from the same cell type as the eQTL–target pair (*P* < 2.2 × 10^−^^16^, chi-square test, degrees of freedom = 25; Supplementary Fig. 3b).

Further analysis focusing on eQTL–target pairs exclusive to individual cell types (CTS eQTL–target pairs) reinforced this observation, demonstrating a similar enrichment of supporting DNA–DNA contacts within corresponding cell types (*P* < 2.2 × 10^−^^16^, chi-square test, degrees of freedom = 25; Fig. 3g). For example, DNA-only contacts linking the *cis* eQTLs of *NELL1* to its promoter were exclusively observed in excitatory neurons, where these eQTLs singularly influence *NELL1* expression variability, and similar cell-specific connections were identified for *PREX2* in oligodendrocytes, where *cis* eQTLs exclusively impact variability in the expression of *PREX2* (Fig. 3h). Together, those cell types exhibiting chromatin contact between a *cis* eQTL and its target gene promoter tend to coincide with those where the *cis* eQTL influences expression of the target gene.

## Cellular variation in XIST–chromosome X contacts

**Fig. 4:**
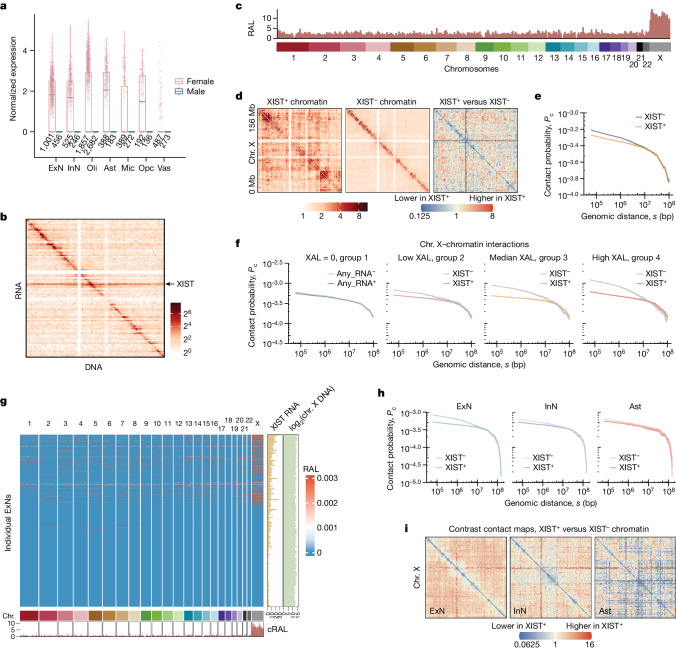
Cellular heterogeneity of XIST–chromatin interactions in female frontal cortex. **a**, XIST expression level of single cells (dots) in each cell type. Box plots are as in Fig. 3e. **b**, RNA–DNA contact map for chromosome X based on ensemble female cells. Each pixel represents the amount of RNA that is transcribed from the genomic bin (row) and is associated with the genomic bin (column). Resolution, 1 Mb. **c**, Distribution of chromatin-associated XIST RNA in female cells. Resolution, 1 Mb. RAL, RNA attachment level. **d**, Chromosome X contact maps for XIST-associated (XIST^+^), XIST-unassociated (XIST^−^) chromatin and their contrast map in female cells. **e**, Frequency of chromatin contacts (*P*_c_) versus genomic distance (*s*) for XIST^+^ and XIST^−^ female chromosome X and chromatin complexes. **f**, Frequency of chromatin contacts versus genomic distance in four groups of female cells with zero, low, medium and high XAL (left to right). The *P*_c_(*s*) curves for chromatin with (Any_RNA^+^) and without (Any_RNA^−^) any associated RNA do not exhibit a marked difference. The difference between XIST^+^ and XIST^−^ *P*_c_(*s*) curves increases as XAL increases. **g**, Genome-wide distribution of XIST–chromatin association in excitatory neurons. Each row corresponds to a single neuron, showing XIST lncRNA attachment levels across genomic regions. Bottom track illustrates the cumulative RNA attachment levels (cRAL) of the XIST lncRNA; right-hand tracks show XIST RNA read count and log-transformed X chromosomal DNA read counts. **h**, Frequency of chromatin contacts versus genomic distance in female excitatory neurons (ExN), inhibitory neurons (InN) and astrocytes (Ast). The difference between XIST^+^ and XIST^−^ *P*_c_(*s*) curves is most pronounced in excitatory neurons. **i**, Contrast contact maps between XIST^+^ and XIST^−^ chromatin on chromosome X in female excitatory neurons, inhibitory neurons and astrocytes.

The XIST lncRNA is detected in female cortical cells but not any male cells (Fig. 4a), which is consistent with its expected presence in female somatic tissues and absence in male tissues^46^. In the ensemble of female cells, XIST lncRNA exhibited a strong association with the entire X chromosome (Fig. 4b,c), consistent with its known ability to spread across one of the X chromosomes (the Xi chromosome)^47,48^.

At the single-cell level, female cortical cells exhibited heterogeneous XAL, as measured by the number of RNA–DNA clusters involving XIST lncRNA and X chromosomal DNA in a nucleus (Extended Data Fig. 9c). Recognizing the potential false negatives, we exercised caution in data analysis and interpretation (Supplementary Note 11 and Extended Data Fig. 9a). Filtering of female cells based on a threshold of total RNA reads per cell (over 5,000) did not eliminate cellular heterogeneity, indicating that the observed heterogeneity cannot be solely attributed to the limited sensitivity of the technique. As expected, the XIST RNA read count in a cell correlated with XAL among female cells (XIST RNA column versus Heatmap, Extended Data Fig. 9e), whereas the number of chromosome X DNA reads remained relatively invariant, confirming that the total DNA read count of the X chromosome is independent of XAL (log_2_(chromosome X DNA column), Extended Data Fig. 9e). Furthermore, the loss of XIST–chromosome X association in single female cells correlates with greater sex difference in gene expression in the human frontal cortex (Supplementary Note 12).

We compared the X chromosomal clusters associated with XIST lncRNA (XIST^+^) and those not associated with XIST lncRNA (XIST^−^). XIST^+^ clusters included RNA–DNA clusters with at least one XIST RNA read whereas XIST^−^ clusters were RNA–DNA and DNA–DNA clusters that did not contain any XIST RNA read. At the chromosomal scale, most DNA–DNA contacts in XIST^−^ clusters were concentrated near the diagonal line in the chromatin contact map (Fig. 4d), similar to the contact maps of autosomes. However, XIST^+^ clusters exhibited not only near-diagonal contacts but also a significant number of contacts spanning distances of 10 Mb or more (Fig. 4d).

Consistent with the chromatin contact maps, the frequency of chromatin contacts (*P*_c_) of XIST^−^ clusters was greater than that of XIST^+^ clusters when the genomic distance (*s*) was less than around 10 Mb (Fig. 4e). To determine whether this separation in *P*_c_(*s*) curves could be attributed to limited sensitivity in detection of XAL at the single-cell level, a stratification analysis was performed. Female cells were stratified into four groups based on zero, low, medium and high XAL. Of note, the zero-XAL group (group 1) contained only XIST^−^ clusters and groups 2, 3 and 4 contained the same number of cells. *P*_c_(*s*) of the XIST^−^ clusters (XIST^−^ *P*_c_(*s*)) was above XIST^+^ *P*_c_(*s*) when the genomic distance was less than about 10 Mb in groups 2, 3 and 4 (Fig. 4f). Importantly, the difference between XIST^−^ and XIST^+^ *P*_c_(*s*) curves increased from group 2 to group 4, indicating that higher XAL in a cell group led to a more pronounced chromatin conformation difference between XIST^−^ and XIST^+^ clusters. As a control, the *P*_c_(*s*) curves of X chromosomal clusters associated with or without any RNA (group 1) were nearly indistinguishable (Fig. 4f). These data suggest that the active X chromosome (Xa) has a higher contact frequency than the inactive X chromosome (Xi) in the sub-10 Mb range of genomic distance in the female human cortex.

Different cell types exhibited different proportions of XAL-positive (XAL^+^) cells (Extended Data Fig. 9b), with excitatory neurons having the highest proportion of these cells (chi-square, *P* = 1 × 10^−16^; Fig. 4g). Considering the greater separation between XIST^+^ and XIST^−^ *P*_c_(*s*) curves (Δ*P*_c_(*s*)) in XAL-high cells, we anticipated seeing a difference in cell type in Δ*P*_c_(*s*), particularly with excitatory neurons exhibiting higher Δ*P*_c_(*s*) compared with other cell types. To test this idea we compared the three cell types with at least 45% of cells exhibiting non-zero XAL, namely excitatory neurons, inhibitory neurons and astrocytes (Extended Data Fig. 9b). As expected, excitatory neurons showed a higher Δ*P*_c_(*s*) and more long-range interactions than inhibitory neurons or astrocytes (Fig. 4h,i). Specifically, the excitatory neuron XIST^+^ *P*_c_(*s*) curve was below that of XIST^−^ when genomic distance was below roughly 10 Mb, but above it at around 10 Mb. Although this transversion was consistently observed for inhibitory neurons and astrocytes, gaps between *P*_c_(*s*) curves were narrower for these cell types. Downsampling excitatory neurons, inhibitory neurons and astrocytes to the same number of cells did not change this observation (Extended Data Fig. 9g). Consistently, sequential fluorescence in situ hybridization analysis showed a larger conformational difference between Xa and Xi in excitatory neurons compared with inhibitory neurons or astrocytes in female mice^49^ (Supplementary Note 13). Together, these data suggest a conserved cell type variation in the spatial organization of the two X chromosomes in the female cortex in both mice and humans.

## Discussion

Human cortical cells exhibited heterogeneity in the distribution of genomic distance-dependent chromatin contact frequency (*P*_c_(*s*)). To capture this diversity, we introduced a metric called the LCS-erosion score, designed to condense *P*_c_(*s*) into a singular value. A higher LCS-erosion score signifies a more pronounced decline in local chromatin contacts. Notably, cells with increased LCS-erosion scores (LCS-eroded cells) demonstrated a tendency towards exhibiting transcriptomic profiles indicative of an ‘older’ cellular age in comparison with their counterparts, LCS-preserved cells. This distinction sheds light on the correlation between the chromatin conformation of a cell and its transcriptomic age, thus extending our understanding from previous observations associating chromatin structural decline with ageing^50^ to the single-cell level. Consequently, we introduce the concept the chromatin conformational age of a cell, as indicated by the LCS-erosion score.

Compared with the male cortex, the female cortex exhibited fewer ‘old’ neurons and more old oligodendrocytes in chromatin conformation age (Fig. 3d and Extended Data Fig. 8d)—that is, a higher oligodendrocyte:neuron ratio in old cells (odds ratio = 6.85, *P* < 2.2 × 10^−^^16^, chi-square test). Compared with male mice, females exhibited more age-related cell deaths in oligodendrocytes but not in neurons^39^. Healthy oligodendrocytes protect normal neuronal activities, and a balance between neurons and oligodendrocytes is required to maintain their bidirectional communications that ensure the necessary protectivity^51,52^. We speculate that the disproportionately greater ratio of old oligodendrocytes in females contributes to explaining the increased risks in neurodegenerative and mental disorders in women.

The genomic sequence at each eQTL usually remains constant across various cell types within an individual, raising questions about why most eQTLs exert their influence on gene expression in specific cell types. Our findings show a connection between cell-type-specific pairing of eQTLs with their target genes and the variability in chromatin contacts between the eQTL and the promoter of the target gene. This underscores the significance of cell type differences in chromatin contacts for future investigations aimed at unravelling this puzzle.

The widespread effect of three-dimensional genome organization on the expression of numerous genes (model 1) and the reciprocal influence of gene expression on chromatin conformation (model 2) continue to be subjects of debate^2,11^. Recent findings suggest that alterations in chromatin conformation precede changes in gene expression during development, lending support to model 1 and challenging model 2 (ref. ^19^). However, experiments involving the degradation of cohesin, a pivotal regulator of chromatin conformation, demonstrated limited impact on the expression levels of most genes in a human cell line, casting doubt on model 1 (ref. ^11^). Our data potentially reconcile the limited impact of cohesin degradation with model 1. The correlation observed between variations in cell-type-specific chromatin contacts and eQTL–target pairing highlights the potential significance of incorporating cell type and interindividual variations to the understanding of a more comprehensive impact.

Following initial debates^53^, chromatin-associated RNA (caRNA) has been increasingly acknowledged as a structural component of chromatin^23^. Work on *Drosophila* and *Gallus gallus* suggested that 2–5% of total chromatin-associated nucleic acids are RNA^54^. In the human MUSIC data, RNA reads account for approximately 4.6% of all chromatin-derived reads, including all DNA–DNA and RNA–DNA clusters, indicating a relatively consistent proportion of RNA in chromatin-associated nucleic acids across species. Interestingly, approximately 11.7% of non-singleton chromatin clusters (DNA–DNA or RNA–DNA) contain RNA reads. Chromatin clusters containing RNA more frequently demonstrate multiplex DNA–DNA contacts than those devoid of RNA, as shown in Extended Data Fig. 4c,d. This observation aligns with the hypothesis that RNA contributes to spatial genome compartmentalization^1,2^.

Women exhibit numerous differences in neurodegenerative diseases and mental disorders compared with men; for example, there are twice as many women with late-onset Alzheimer’s disease than men, and women have a significantly higher frequency of adulthood depression and anxiety. Notably, many X-linked genes are expressed in the brain and have a role in cognitive functions^55^. MUSIC data showed that, in female cortical cells, the diminishing association between XIST and chromosome X correlates with reduced structural differences between active and inactive X chromosomes, and this is associated with greater differences in chromosome X gene expression between the sexes. These multimodal, single-cell data provide a critical resource for future investigations of sex differences in health and disease. In summary, MUSIC provides a unique tool for joint analysis of gene expression, multiplex chromatin interactions and RNA–chromatin associations at single-cell resolution from complex tissue.

## Methods

### Critical reagents

#### RNA linker

**Extended Data Fig. 2:**
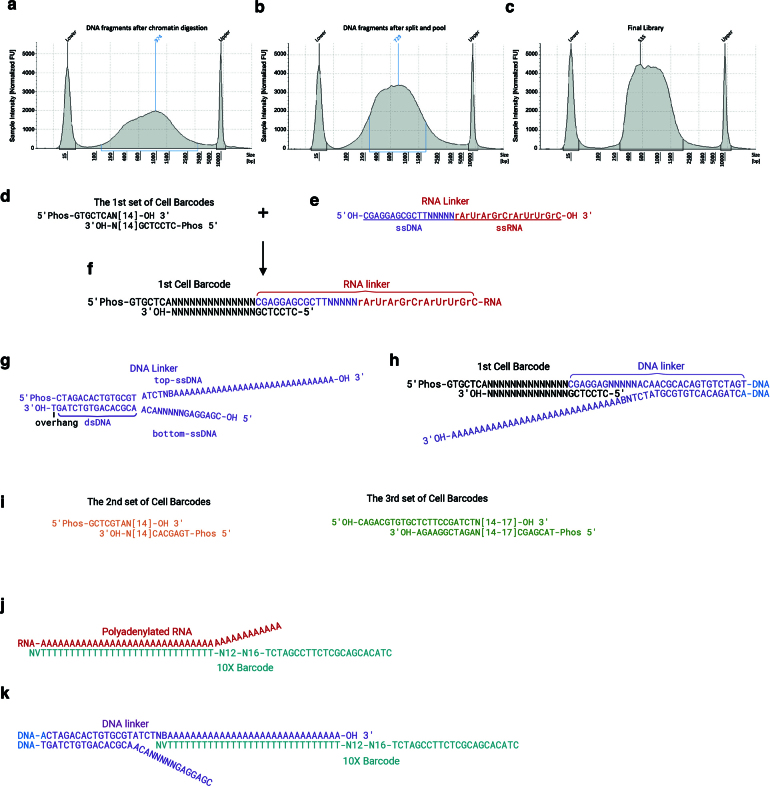
Critical reagents and reactions. (a-c) DNA size distribution of DNA fragments (a) after chromatin digestion, corresponding to Fig. 1a, (b) after three rounds of cell barcode ligation, corresponding to Extended Data Fig. 1e, and (c) in the final library, corresponding to Extended Data Fig. 1h. (d) the 1st set of Cell Barcodes. (e) The RNA Linker with half single strand RNA (ssRNA, red) and half single strand DNA (ssDNA, purple) (f) Base-pairing assisted ligation of the ssDNA (purple) in the RNA Linker with the 1st set of Cell Barcodes (black). (g) The Y-shaped DNA Linker with a double strand region (dsDNA) and single strand regions (top-ssDNA and bottom ssDNA) with a 3’dT overhang. (h) The DNA Linker (purple) is ligated with 3’ dA-tailed double-stranded genomic DNA (blue) and with the 1st set of Cell Barcodes (black). (i) the 2nd and the 3rd sets of Cell Barcodes. The 3rd set of Cell Barcodes contains a fraction of Illumina Read2 adapter sequence. (j) Hybridization of the RNA’s polyA tail (red) with the poly(dT) region of the 10X Barcodes (turquoise). (k) Hybridization of the top-ssDNA of the DNA linker (purple) with the poly(dT) region of the 10X Barcodes (turquoise).

**Extended Data Fig. 3:**
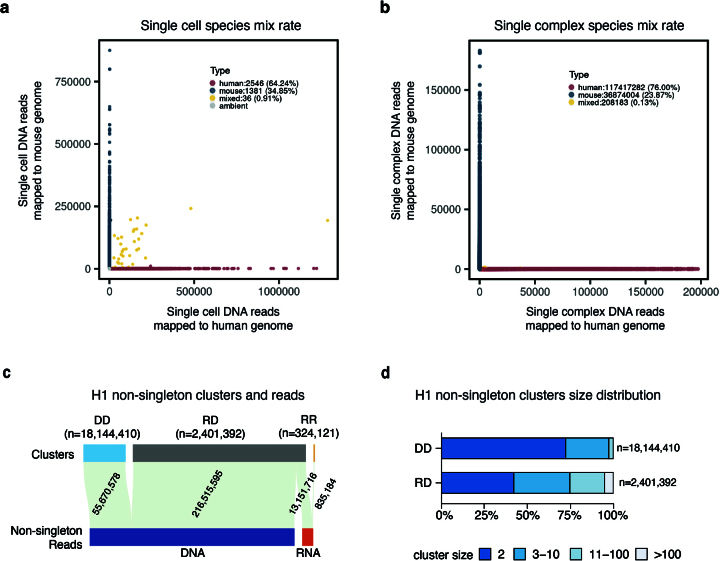
Mixed-species analysis. (a) The cells (dots) with more than 1000 reads (read count filter = 1000) are colored coded to red (human), blue (mouse) if 95% or more of the reads are mapped to a single species (purity filter = 95%), or yellow (mixed) if otherwise. The cells with less than 1000 reads (gray dots, ambient) are not used in the calculation of mixed-species rate. (b) The complexes (dots) are colored coded to red (human), blue (mouse) when 99% or more of the reads are mapped to a single species, or yellow (mixed) if otherwise. The complexes with 0 - 200000 DNA reads are plotted. (c) The distribution of DNA reads and RNA reads in DNA-DNA (DD), RNA-DNA (RD), and RNA-RNA (RR) clusters. (d) Cluster size distributions of DD and RD clusters.

**Extended Data Fig. 4:**
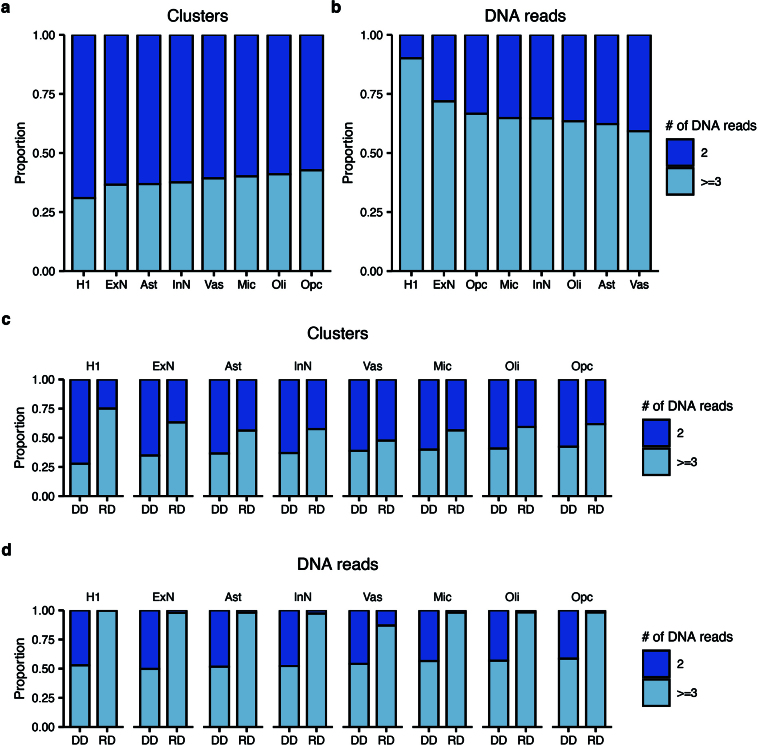
Proportions of pairwise or multiplex interactions. The proportions of clusters (a) and DNA read counts (b) that consist of multiplex chromatin interactions (DNA reads >=3, light blue) in each cell type. Both RD and DD clusters are included in this analysis. (c) Proportions of clusters with pairwise (2 DNA reads, dark blue) or multiplex chromatin interactions (3 or more DNA reads, light blue) in DNA-only (DD) and RNA-DNA (RD) clusters in each cell type. (d) Proportions of DNA read counts with clusters corresponding to pairwise (dark blue) or multiplex (light blue) chromatin interactions.

**Extended Data Fig. 5:**
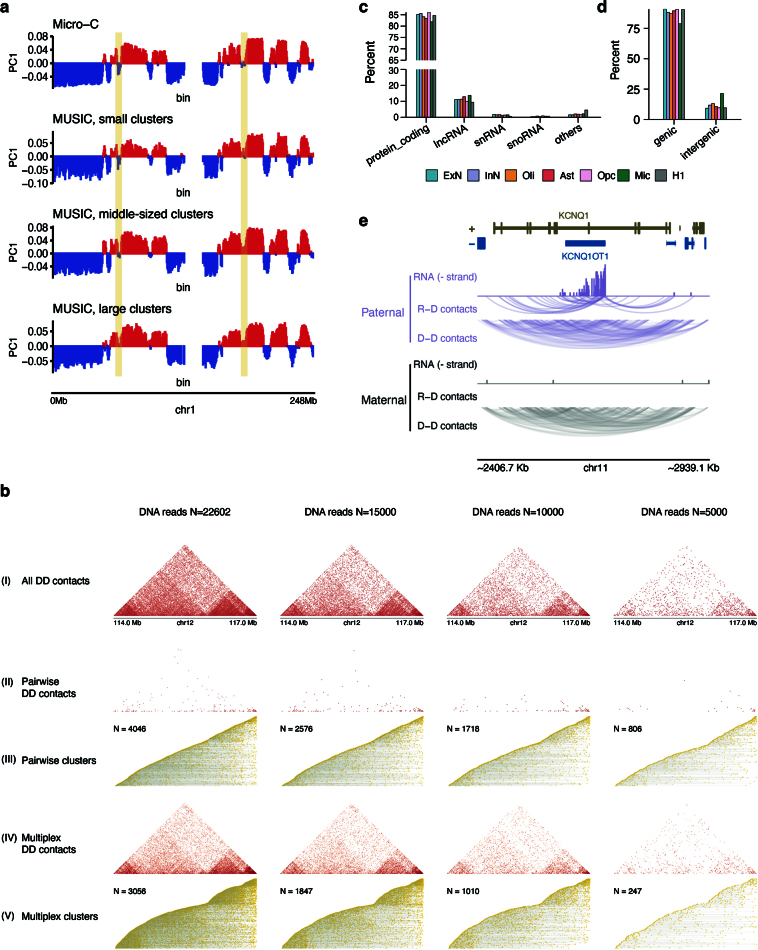
Chromatin contacts and RNA in H1 cells. (a) Compartment scores (PC1) across the entire Chromosome 1 at 500 Kb resolution. PC1s are calculated from Micro-C, MUSIC’s small clusters (2–10 DNA reads), middle-sized clusters (10–50 DNA reads), and large clusters (50–100 DNA reads). Positive and negative PC1s correspond to A and B compartments. Two small B compartments in MUSICs’ small clusters became indistinguishable in the larger clusters (vertical yellow stripes), corresponding to slightly fewer A/B compartment switches in the small clusters. (b) Downsampling the original 22,602 UMNDBC DNA reads mapped to this region (Chr12:114-117 Mb) to 15,000, 10,000, and 50,000 reads (columns). Rows from the top to the bottom are 2D contact map of all DD contacts that include multiplex and pairwise contacts (I), 2D contact map of pairwise DD contacts (II), a cumulative view of every DD cluster in pairwise complexes (III), 2D contact map of multiplex DD contacts (IV), and a cumulative view of every DD cluster in multiplex complexes (V). (c) The proportions of each type of RNA species (y axis) in each cell type (color coded). (d) The proportions of genic and intergenic RNA reads in each cell type. (e) Genome track view of the RNA reads (RNA), RNA-DNA (R-D) contacts, and DNA-DNA (D-D) contacts in the paternal (top) and maternal (bottom) allele in H1 cells. All the RNA reads shown are those mapped to the KCNQ1OT1 gene in the strand consistent with the direction of transcription of the KCNQ1OT1 gene (- strand).

**Extended Data Fig. 6:**
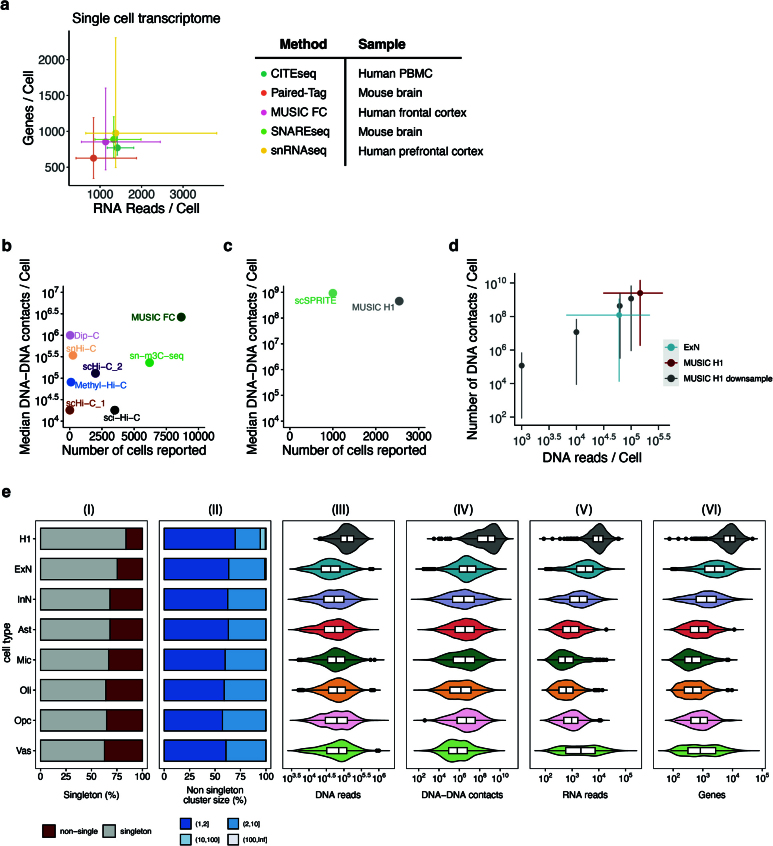
Comparison of technologies. (a) The number of RNA reads per cell (x axis) vs. the number of detected genes per cell (y axis) of CITE-seq, SNARE-seq, PairedTag, snRNA-seq, SNARE-seq and MUSIC frontal cortex data (MUSIC FC). The central dot represents the average number and the bars indicate the first and the third quartile of the distribution of all the cells (x axis) and per cell total detected genes (y axis). (b-c) The number of cells (x axis) vs. the median number of DNA-DNA contacts per cell (y axis) for each technique. For MUSIC and scSPRITE, the number of pairwise DNA-DNA contacts are decomposed from multiplex clusters. (d) Downsampling of the sequencing reads of the H1 library. Excitatory neuron (ExN) is used as an example cortical cell type to compare with the downsampled H1 data. The number of chromatin contacts per cell (y axis) is plotted against the number of DNA reads per cell (x axis). The mean (dot) and 95% range of the distribution (whiskers) of the original H1 (red), the downsampled H1 data (gray), and ExN (blue). (e) Summary of MUSIC data by cell type. (I) The proportions of singleton (gray) and non-singleton clusters (red). (II) Cluster sizes of the non-singleton clusters. (III-VI) The distributions of the numbers of DNA reads, DNA-DNA contacts, RNA reads, and detected genes in the single cells. Sample sizes for H1, Ex, In, Ast, Mic, Oli, Opc, Vas are 2267, 1457, 771, 571, 661, 4539, 328 and 760 respectively. For the middle boxplot, the left, center and right edge represent the 25th percentile, median and 75th percentile, respectively. Whiskers extend to 1.5 times the Interquartile Range from the box edges. Data points beyond the whiskers are outliers.

**Extended Data Fig. 7:**
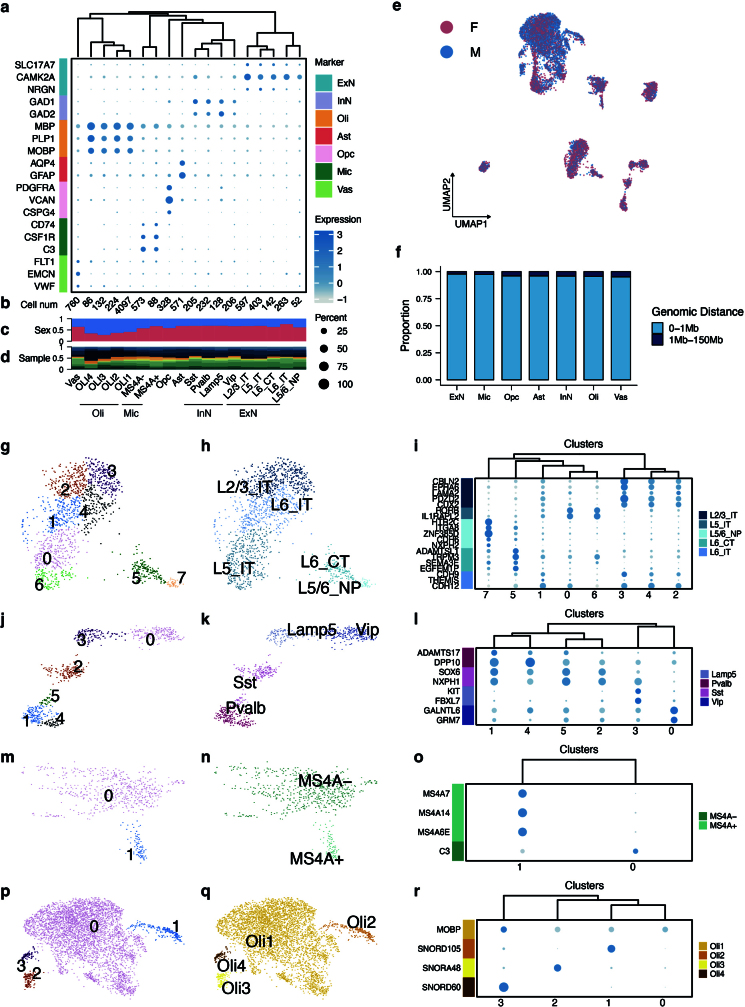
MUSIC analysis of human frontal cortex. Marker gene expression (a), number of cells (b), percent of female (pink) and male cells (blue) (c), and the relative proportion of each sample (d) in every single-cell cluster, and the assigned cell type and subtype for each cluster. Assigned subtypes and marker gene expression in excitatory neurons (g-i), inhibitory neurons (j-l), microglia (m-o), and oligodendrocytes (p-r). (e) Female (pink) and male (blue) cells in the UMAP embedding. (f) Proportions of single cells (y axis) with their most frequent chromatin interactions in each genomic bin (0-1 Mb, 1Mb-150Mb) (x axis) in each cell type.

**Extended Data Fig. 8:**
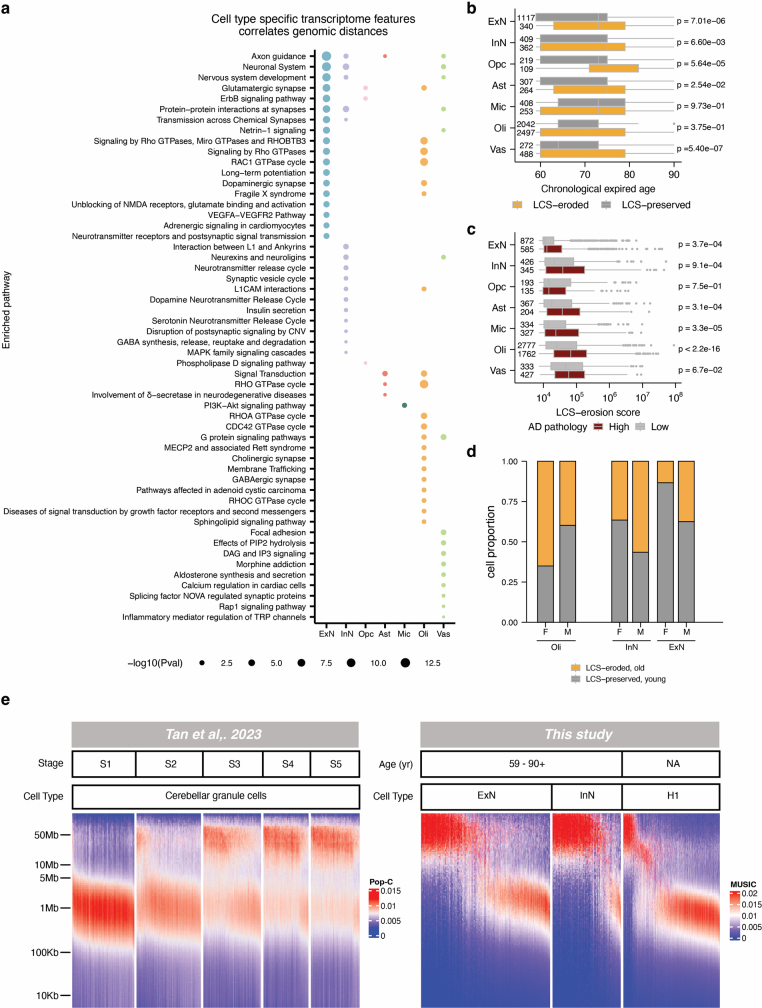
Correlation between Pc(s) and transcriptomic age among single cells. (a) The enriched pathways (row) in those genes that exhibit a correlated expression level with the LCS-erosion score across all the single cells in each cell type (column). The size of the dot represents the enrichment score (-log10(P value)) with larger dots indicating higher significance. (b) Distribution of the chronological ages (age at death, y axis) in the LCS-preserved (gray) and LCS-eroded cells (orange) in each cell type (column). Wilcoxon test, One-sided. Left numbers: sample size. Boxplot: the right edge, center line and left edge represent the 75th percentile, median and 25th percentile, respectively. Whiskers extend to 1.5 times the Interquartile Range from the box edges. Data points beyond the whiskers are outliers. (c) LCS-erosion scores (y axis) in the cells within the samples with high (Braak stage >=4) and low (Braak stage <3) AD pathology. Wilcoxon test, One-sided. Left numbers: sample size. Boxplot definition is the same as (e). (d) Proportion of LCS-eroded cells and LCS-preserved cells by gender (female (F), male (M)) within oligodendrocytes (Oli) and Neurons (InN, ExN). (e) Number of contacts (color) plotted against genomic distance (y-axis) in single nuclei (columns) using data from Tan et al. (left) and our study (right). Rows represent genomic bins with exponentially increasing sizes. The two datasets were plotted using the same computational program and genomic bin sizes. The ‘Stage’ labels correspond to cell groups identified by Tan et al., where Stages S1 to S5 are enriched with cells of chronological ages 0.2, 1, 10, 30, and 80, respectively. ExN: Frontal cortex excitatory neurons. InN: Frontal cortex inhibitory neurons.

**Extended Data Fig. 9:**
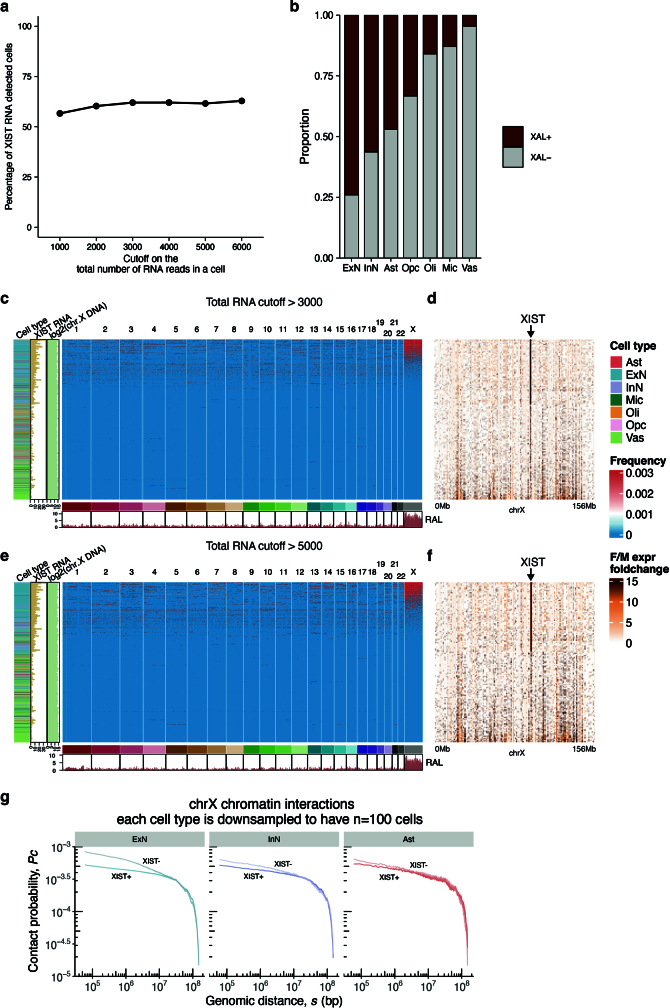
XIST-chromatin association in single female cells. (a) The change of the percentage of XIST lncRNA detected cells (y axis) in the female cortical cells that satisfy the threshold on the total number of RNA reads in a cell (x axis). (b) The variation of the proportions of the observed XAL+ cells across cell types (columns) in the female cortex under total RNA larger than 5000 cutoff. (c-f) The female cells are filtered so that every cell has at least 3000 RNA reads (c-d) or at least 5000 RNA reads (e-f). (c, e) Genome-wide distribution of XIST-chromatin association in individual female cortical cells. Each row represents a single cell. The RNA attachment level (RAL) of the XIST lncRNA (intensity of red color) at any genomic region on any chromosome is plotted with the corresponding genomic coordinates (x axis). Resolution: 1 Mb. Track at the bottom: cumulative RAL of XIST. Tracks on the left indicate the cell type (color), XIST RNA read count (XIST RNA), X chromosomal DNA read counts (chr.X DNA) in log2 scale. (d, f) sex-fold-change of a previously identified gene with incomplete X chromosome inactivation (XCI) (column) between this female cell (row) and the average expression of the male cells of the matched cell type. Only the genes expressed in at least 20 cells are plotted. (g) Downsampling analysis of cell numbers. X chromosome Pc(s) curves in excitatory neurons (ExN), inhibitory neurons (InN), and astrocytes (Ast) are plotted from the same number of female cells (n = 100). The difference between XIST+ (darker color) and XIST- Pc(s) (lighter color) curves is most pronounced in ExN.

The RNA linker is a single-stranded chimeric oligonucleotide with 17 DNA nucleotides at its 5′ end (ssDNA; Extended Data Fig. 2e) and 10 RNA nucleotides at its 3′ end (ssRNA; Extended Data Fig. 2e), denoted as 5′-/5OH/CGAGGAGCGCTTNNNNNrArUrArGrCrArUrUrGrC/3OH/-3′, where A, C, G, T and N denote DNA nucleotides, rA, rC, rG and rT denote RNA nucleotides and NNNNN denotes five randomized DNA nucleotides that serve as a UMI. The RNA linker was synthesized by Integrated DNA Technologies (IDT).

The RNA linker is designed for efficient ligation with (1) RNA through the RNA linker ssRNA, and (2) the first set of cell barcodes through the RNA linker ssDNA, which is complementary to the seven nucleotide (nt) overhang in the first set of cell barcodes.

#### DNA linker

The DNA linker is a hybridized product of two DNA strands, with the top strand being 5′-/5Phos/CTAGACACTGTGCGTATCTNBAAAAAAAAAAAAAAAAAAAAAAAAAAAAAA/3OH/-3′, where N denotes a random base and B denotes any base except A, and with the bottom strand being 5′-/5OH/CGAGGAGNNNNNACAACGCACAGTGTCTAGT/3OH/-3′, where NNNNN denotes five randomized DNA bases that serve as a UMI. Following hybridization, the DNA linker contains 15 bp double-stranded DNA and 36 nt (top ssDNA) and 15 nt unhybridized ssDNA (bottom ssDNA), and a single base (T) overhang at the bottom strand (Extended Data Fig. 2g). The 36 nt top ssDNA is reverse complementary to 10X barcodes in the Chromium Next GEM Single Cell 3′ Reagent kit (PN-1000268). The two strands of the DNA linker were synthesized by IDT.

The DNA linker is designed for (1) efficient ligation with fragmented chromosomal DNA through the 1 nt overhang of the DNA linker by a sticky-end ligation, (2) efficient ligation with cell barcodes through the 15 nt bottom ssDNA of the DNA linker, and (3) efficient hybridation with 10X barcodes through the polyA sequence within the top ssDNA.

#### Cell barcodes

The cell barcodes contain three sets of barcodes referred to as the first, second and third sets. Every set of cell barcodes has three components, namely a 7 nt top-strand overhang, a 14 bp dsDNA region and a bottom-strand overhang (7 nt for the first and second sets, 11 nt for the third). The 14 bp dsDNA region contains a unique sequence to every cell barcode (double-stranded N14; Extended Data Fig. 2d,i). Every set of cell barcodes contains 96 unique barcodes, each being unique in this 14 bp dsDNA (Supplementary Table 2).

In the current version of MUSIC (v.1.0), each set of cell barcodes contains 96 unique barcodes based on their dsDNA regions, resulting in a total of 884,736 unique sequence combinations. The three sets of cell barcodes are designed to maximize ligation efficiency for sequential ligation of the first set of cell barcodes with the RNA and DNA linkers, the second set with the first set, and the third set with the second set. Optimal ligation efficiency is achieved by the complementarity of the overhang sequences (Extended Data Fig. 2d,i). Out-of-order ligations, such as that of the third set with the first set of cell barcodes, are minimized because the overhang of the third set does not complement with that of the first set.

In addition, the third set of cell barcodes is also designed to complement the 22 nt sequence at the 3′ side of the index adaptors.

The first and second sets of cell barcodes were synthesized by Sigma-Aldrich and the third by IDT.

#### 10X barcodes

The 10X barcodes are included in the Chromium Next GEM Single Cell 3′ Reagent kit (PN-1000268) from 10X Genomics. Each 10X barcode is an 82 nt oligonucleotide with a partial (22 nt) Illumina Read1 sequence (Read1), a 16 nt unique barcode sequence (N16, 10X GEM barcode), a 12 nt UMI (N12, 10X UMI), a 30 nt polyT sequence, a V (A, C or G) and an N (any base) (10X barcode; Extended Data Fig. 2j, k). The 10X GEM barcode is shared among the barcodes of the same GEM; the 10X UMI is unique to every 10X barcode.

#### Index adaptors

The index adaptors contain three segments, namely the 24 nt Illumina P7 sequence, an 8 nt unique identifier sequence called I7 and the 34 nt Illumina Read2 sequence (Supplementary Table 2). In this release, MUSIC v.1.0 uses eight distinct I7 barcodes providing a total of approximately 83.5 million complex barcodes (8 (I7 barcodes) × 3.5 million (10X barcodes)). Eight index adaptors are used for each library construction, each of which has a unique I7 sequence. We call these eight index adaptors a set of index adaptors, each of which can hybridize with the complementary read2 sequence in the third set of cell barcodes to initiate a PCR reaction.

Meanwhile these serve as sample barcodes. We designed a total of three sets of index adaptors to allow for the construction of three libraries from three input samples and sequencing them together (Supplementary Table 2). These three sets of index adaptors share P7 and read2 sequences and differ by their I7 sequences; they also serve as a sample index to differentiate the three samples. The index adaptors were synthesized by IDT.

#### Universal adaptor

The universal adaptor contains an Illumina P5 sequence and an Illumina read1 sequence (Supplementary Table 2). The universal adaptor can hybridize with 10X barcodes through their complementary read1 sequence to initiate a PCR reaction. The universal adaptor was synthesized by IDT.

#### Cell culture

H1 human embryonic stem cells and E14 mouse embryonic stem cells were obtained from the 4D Nucleome Consortium and cultured according to 4D Nucleome Consortium-approved protocols (https://www.4dnucleome.org/). In brief, H1 cells were grown at 37 °C under 5% CO_2_ on Matrigel (Corning, 354277)-coated dishes. Cells were maintained in complete mTeSR medium prepared from basal medium (Corning, 85851) with 5× supplement (Corning, 85852). Medium was replaced daily. Cell passage numbers were kept below P10. E14 cells were cultured on plates coated with 0.1% gelatin (EMD, SF008) in serum-free 2i/LIF medium; this medium was made from base medium (1:1 mixture of NeuroBasal medium (Gibco, 21103-049) and DMEM/F12 medium (Gibco, 11320-033) supplemented with 0.5× N2 supplement (Gibco, 17502-048), 0.5× B27 supplement (Gibco, 17504-044) and 0.05% bovine serum albumin (BSA) fraction V (Gibco, 15260-037)), supplemented with 1 µM PD0325901 (Reprocel, 04-0006-02C), 3 µM CHIR99021 (Reprocell, 04-0004-02C), 0.15 mM monothioglycerol (Sigma, M6145-25ML) and 1,000 U ml^−1^ LIF (Cell Guidance Systems, GFM200). Medium was replaced daily, and cell passage number was kept below P10.

#### Crosslinking and nuclei isolation for cell lines

After cells had become confluent in a 10 cm dish, medium was removed and washed once with PBS. Accutase (1 ml; EMD, SF006) was added, with incubation for 3 min at 37 °C to dissociate cells. PBS (10 ml) was used to generate a single-cell suspension by pipetting. Cell pellets were formed by centrifugation at 330*g* for 3 min. Next, 10 ml of 2 mM disuccinimidyl glutarate (DSG) dissolved in PBS was added to crosslink and resuspend the cells in a LoBind tube, with incubation at room temperature for 45 min under gentle rotation. Following incubation, cells were collected by centrifugation at 1,000*g* for 4 min to remove DSG solution, washed once with PBS and then centrifuged again at 1,000*g* for 4 min to remove supernatant. Following washing, cells were thoroughly resuspended in 10 ml of PBS containing 3% formaldehyde and incubated for 10 min with gentle rotation. The crosslinking reaction was stopped by the addition of 3 ml of 2.5 M glycine per 10 ml of 3% formaldehyde, with incubation for 5 min with rotation. Cells were then centrifuged at 1,000*g* for 4 min to remove supernatant. Next, cells were washed twice with ice-cold PBS containing 0.5% BSA (w/v) and centrifuged at 1,000*g* for 4 min. Following this wash, cells were resuspended in PBS with 0.5% BSA (w/v), each cell aliquot then containing 5 million cells in a 1.5 ml tube. Cell pellets were obtained by centrifugation at 1,000*g* for 5 min, snap-frozen in liquid nitrogen and stored at −80 °C.

Frozen cells were thawed on ice and resuspended in 1.4 ml of cell lysis buffer A for every 5 million cells, as previously described^12^ (50 mM HEPES pH 7.4, 1 mM EDTA pH 8.0, 1 mM EGTA pH 8.0, 140 mM NaCl, 0.25% Triton X-100, 0.5% IGEPAL CA-630, 10% glycerol,  proteinase inhibitor cocktail). For the mixed-species experiment, equal amounts of human H1 (2.5 million) and mouse E14 (2.5 million) cells were resuspended together. Following 10 min incubation on ice, cell pellets were collected by centrifugation at 900*g* for 4 min at 4 °C. Cell pellets were then resuspended in 1.4 ml of cell lysis buffer B (10 mM Tris-HCl pH 8.0, 1.5 mM EDTA, 1.5 mM EGTA, 200 mM NaCl and proteinase inhibitor cocktail) and kept on ice for 10 min. The nuclei thus isolated were collected at 900*g* for 5 min at 4 °C. Two hundred microlitres of rCutSmart buffer (NEB, B7204S) containing 0.25% SDS was used to thoroughly resuspend and permeabilize the nuclei, with incubation at 62 °C for 10 min with Eppendorf Thermomixer C. Following incubation, 60 µl of rCutSmart buffer containing 10% Triton X-100 (w/v) was mixed with the SDS solution and the reaction incubated at 37 °C for 15 min while shaking at 800 rpm. Treated nuclei were centrifuged at 900*g* for 2 min at 4 °C to remove supernatant and washed once with rCutSmart buffer.

#### Crosslinking and nuclei isolation from postmortem brain

Each 50 mg of postmortem human brain frontal cortex sample was kept on ice in a 1.5 ml LoBind tube and chopped into smaller pieces by pestle. Brain samples were transferred into a 15 ml LoBind tube and incubated at room temperature for 45 min with gentle rotation in 10 ml of 2 mM DSG dissolved in PBS. Following incubation, tissue samples were centrifuged at 1,000*g* for 4 min and washed once with PBS to remove DSG solution. Following washing, samples were thoroughly resuspended in 10 ml PBS containing 3% formaldehyde and incubated for 10 min with gentle rotation. The crosslinking reaction was stopped by the addition of 4 ml of 1.25 M glycine, followed by incubation for 5 min with rotation. Samples were then centrifuged at 1,000*g* for 4 min and washed twice with ice-cold PBS containing 0.3% BSA (w/v).

A Chromium Nuclei Isolation kit (10X Genomics, 1000494) was used to isolate nuclei from crosslinked cortex samples according to the manufacturer’s user guide). Specifically, 50 mg of frozen tissue was placed in a prechilled sample-dissociation tube, then 400 µl of the lysis buffer provided was added to the tube and tissues were dissociated until homogeneous using a plastic pestle. Next, 600 µl of lysis buffer was added to the tube and the contents mixed ten times by pipetting. Following 10 min incubation on ice, the solution was evenly loaded into two nuclei isolation columns and centrifuged at 16,000*g* for 20 s at 4 °C. The flowthrough in the collection tube containing the nuclei was vortexed for 10 s at 3,200 rpm to resuspend nuclei. The collection tube was then centrifuged for 3 min at 500*g* and 4 °C to pellet the nuclei and the supernatant was removed. The nuclei were resuspended in 500 µl of debris removal buffer provided with the kit by pipetting 15 times, then centrifuged at 700*g* for 10 min at 4 °C and the supernatant removed. The nuclei were resuspended twice in 1 ml of wash and resuspension buffer. The supernatant was removed following centrifugation at 500*g* for 5 min at 4 °C, which left a purified pellet of isolated nuclei (Supplementary Fig. 1a,b). All the following steps were identical for both cell lines and human cortex samples.

#### Ligation of the RNA linker with RNA

Nuclei were resuspended in 250 µl of 5′ phosphorylation master mix (T4 PNK buffer, 500 U ml^−1^ T4PNK, 1 mM ATP, 1 U µl^−1^ RNAse inhibitor (Roche, 3335399001)) with incubation at 37 °C while rotating at 800 rpm for 1 h to phosphorylate the 5′ ends of RNA. Nuclei were washed once with PBS wash buffer 1 (PBS, 1 mM EDTA, 1 mM EGTA and 0.1% Triton X-100) and three times with PBS wash buffer 2 (PBS, 0.5% BSA (w/v) and 0.1% Triton X-100). The RNA linker is a single-stranded chimeric oligo with the DNA 5′ hydroxyl group end and the RNA 3′ hydroxyl group (5′-OH-CGAGGAGCGCTTNNNNNrArUrArGrCrArUrUrGrC-OH-3′). A RNA ligation mix was made with 4 µM RNA linker, T4 RNA ligation buffer, 400 U ml^−1^ T4 RNA ligase 1, 15% PEG 8000, 1 mM ATP and 1 U µl^−1^ RNAse inhibitor. Isolated nuclei were thoroughly mixed with 250 µl of the RNA ligation mix to ligate the RNA linker with nuclear RNA. The mixture was incubated at 25 °C for 2 h then at 16 °C overnight, with an intermittent mixing at 800 rpm (30 s on and 270 off). Following ligation, nuclei were washed once with PBS wash buffer 1 and three times with PBS wash buffer 2.

#### Chromatin digestion

All washed nuclei were resuspended in a digestion master mix (300 µl of rCutSmart buffer containing 30 µl of 5,000 U ml^−1^ HpyCH4V with 1 U µl^−1^ RNAse inhibitor). This master mix was kept for 3 h at 37 °C while rotating at 800 rpm. Nuclei were collected at 900*g* for 2 min with the supernatant removed. Nuclei were further washed once with 900 µl of PBS wash buffer 1 and three times with 900 µl of PBS wash buffer 2.

#### Ligation of the DNA linker with DNA

To create the sticky end for DNA linker ligation, the nuclei was suspended in 250 µl of dA-tailing reaction master mix (NEBNext dA-tailing reaction buffer, 200 U ml^−1^ Klenow fragment, 1 U µl^−1^ RNAse inhibitor) and incubated at 37 °C while rotating at 800 rpm for 1.5 h. Next, nuclei were washed once with PBS wash buffer 1 and three times with PBS wash buffer 2. The DNA linker is a hybridized product of two DNA strands, with the top strand being 5′-Phos-CTAGACACTGTGCGTATCTNBAAAAAAAAAAAAAAAAAAAAAAAAAAAAAA-OH-3′ and the bottom-strand 5′-OH-CGAGGAGNNNNNACAACGCACAGTGTCTAGT-OH-3′. The DNA linker contains 14 bp dsDNA, 36 nt top ss DNA and 15 nt bottom ssDNA (Extended Data Fig. 2g,h). A DNA ligation master mix comprised 4.5 µM DNA linker, 0.2× of 2× Instant Sticky-end Ligase Master Mix (NEB, M0370), 0.8× of 5× Quick Ligase Buffer (NEB, B6058S), 6% (v/v) 1,2-propanediol (Sigma-Aldrich, 398039) and 1 U µl^−1^ RNAse inhibitor). To ligate the DNA linker with the sticky-end DNA, all nuclei were thoroughly mixed with 250 µl of DNA ligation master mix. The ligation reaction was carried out at 20 °C for 6 h with an intermittent mixing at 1,600 rpm (30 s on and 270 s off).

#### Ligation of cell barcodes

To phosphorylate the 5′ end of the linker, nuclei were resuspended in 250 µl of 5′ phosphorylation master mix and incubated at 37 °C while rotating at 800 rpm for 1 h. Nuclei were then washed once with PBS wash buffer 1 and three times with PBS wash buffer 2. Nuclei were resuspended in 900 µl of PBS wash buffer 2 with 0.2 U µl^−1^ RNase inhibitor and filtered through a 10 µM cell strainer (pluriStrainer, 43-10010-50). Six microlitres of the nuclear suspension was stained with 6 µl of ethidium homodimer-1, and nuclei counted using a Countess II Automated Cell Counter (ThermoFisher). A total of 288 barcodes were taken from Hawkins et al.^56^ and split into sets 1, 2 and 3. Each barcode takes the form of 7 nt_overhang-dsDNA-7 nt_overhang (Extended Data Fig. 2d,i and Supplementary Table 2). Those 288 barcodes collected were then termed the cell barcodes. Three ligation master mixes were prepared, each containing one set of barcodes (5.4 µM), 0.2× of 2× Instant Sticky-end Ligase Master Mix, 0.8× of 5× Quick Ligase Buffer, 6% (v/v) 1,2-propanediol and 0.8 U µl^−1^ RNAse inhibitor. The ligation master mixes were named mixes 1, 2 and 3, corresponding to barcode sets 1, 2 and 3, respectively.

##### First round of split-pooling

Up to 100,000 nuclei were collected for split-pooling to ensure that the majority were labelled with unique cell barcode combinations. The nuclear suspension was made up to 1,144 µl with PBS wash buffer 2 and 24 µl of RNAse inhibitor and subsequently split into 96 wells. To ligate barcode set 1 with the RNA and DNA linkers, nuclei in each well are incubated overnight in ligation master mix 1 at 20 °C with an intermittent mixing at 1,600 rpm (30 s on and 270 off). Following overnight incubation the reaction was quenched by the addition of 60 µl of quenching buffer (PBS, 50 mM EDTA, 50 mM EGTA, 0.1% Triton X-100) and incubated for 10 min at 20 °C. Nuclear solutions from the 96 wells were pooled into a 15 ml LoBind tube; 95 µl of quenching buffer was then added to each well to rinse and collect any remaining nuclei, with pooling into the same 15 ml tube. Nuclei were centrifuged at 900*g* for 4 min and then transferred to a 1.5 ml tube with 0.5 ml of the remaining supernatant. PBS wash buffer 2 (500  µl) was used to rinse the 15 ml tube and collect residual nuclei into the same 1.5 ml tube. Nuclei were washed three times with 900 µl of PBS wash buffer 2 by centrifugation at 900*g* for 2 min.

##### Second and third rounds of split-pooling

Pooled nuclei were subjected to the same split-pooling procedure as in the first round, except that ligation master mixes 2 and 3 were used for the second and third rounds, respectively, replacing ligation master mix 1.

#### Addition of complex barcodes

We used a combination of two sets of barcodes to jointly differentiate individual molecular complexes, referred to as the complex barcodes. The first set of barcodes are those 3.5 million oligos provided in the Chromium Next GEM Single Cell 3′ Reagent kit (PN-1000268; 10X barcodes). Each oligo is an 82-base oligonucleotide with a 16 nt barcode and 12 nt UMI (10X BC + UMI; Extended Data Fig. 2j,k). The second set of barcodes is composed of eight barcodes (index barcodes), each being 8 nt (I7 in Extended Data Fig. 1h and Supplementary Table 2).

The nuclei were resuspended in 250 µl of 3′ dephosphorylation buffer (PNK buffer, 0.5 U µl^−1^ T4PNK, 1 U µl^−1^ RNAse inhibitor) and incubated at 37 °C for 1 h, with rotation at 800 rpm, to convert any 2′, 3′ cyclic phosphate on RNA to 3′-OH. Nuclei were washed once with PBS wash buffer 1, three times with PBS wash buffer 2 and centrifuged at 900*g* for 2 min. To add polyA sequences to all RNA molecules, nuclei were resuspended in polyA tailing buffer (*E. coli* poly(A) polymerase reaction buffer, 0.08 U µl^−1^ *E. coli* poly(A) polymerase, 1 mM ATP, 1 U µl^−1^ RNase inhibitor). The mixture was kept at 37 °C for 10 min while rotating at 800 rpm. Following the addition of polyA tails, nuclei were thoroughly resuspended in PBS with 0.04% BSA (w/v) and filtered through a 10 µM cell strainer (pluriStrainer, 43-10010-50) into a 1.5 ml tube to obtain isolated nuclei. Six microlitres of the nuclei-containing solution was stained with 6 µl of ethidium homodimer-1, and nuclei counted using a Countess II Automated Cell Counter. Five thousand single nuclei were transferred to a Covaris microtube-15, which was then filled to 15 µl with PBS and 0.04% BSA (w/v). Nuclei were sonicated using a Covaris M220 Focused-ultrasonicator (water temperature 6 °C, incident power 50 W, duty factor 5) for 5 min to release chromatin complexes.

To add 10X barcodes to polyadenylated RNA and the top ss end of the DNA linker, sonicated nuclei complexes were transferred into a 1.5 ml LoBind tube and mixed with 25 µl of water, 18.8 µl of reverse transcription (RT) reagent B, 2 µl of reducing agent B and 8.7 µl of RT enzyme C. The mixture was transferred to one well in chromatin immunoprecipitation sequencing G, which was then loaded onto the 10X Chromium controller according to steps 1.1–1.5 in the protocol of the Chromium Next GEM Single Cell 3′ Reagent kit. The retrieved droplets were transferred to a PCR tube for complementary DNA synthesis according to the 10X protocol. The droplets were dispersed, with the aqueous phase obtained according to step 2.1 in the 10X protocol.

The aqueous phase containing nucleic acids was filled to 200 µl with nuclease-free water and split into eight aliquots in LoBind 1.5 ml tubes; next, 25 µl of 2× reverse crosslinking buffer (400 mM NaCl, 0.4% SDS, 50 mM EDTA, 50 mM EGTA, 0.04 U µl^−1^ proteinase K) was added to each tube and the ensuing reverse crosslinking reaction was incubated at 50 °C for 2 h, then at 55 °C overnight, with shaking at 800 rpm. In each aliquot the reverse crosslinked nucleic acids were purified using the Monarch RNA purification kit (NEB, 76307-460) with elution into 21 µl of nuclease-free water. Eluted DNA and RNA molecules were incubated at 55 °C for 15 min with isothermal amplification buffer II, 0.32 U µl^−1^ Bst 3.0 DNA polymerase, an additional 6 mM MgSO_4_, 1.4 mM dNTP Mix and 0.5 U µl^−1^ RNasin. The product was purified with 1.8× RNA clean Ampure beads (Beckman Coulter Life Science, A63881) and eluted into 20 µl of nuclease-free water. PCR was performed for each aliquot with 2.5 µl of 10 µM shared Universal Adaptor (P5 and read1 in Extended Data Fig. 1h) and 2.5 µl of 10 µM aliquot-specific primer in 25 µl of NEBNext Ultra II Q5 Master Mix. The aliquot-specific primers are the eight index adaptors (‘Critical reagents’). PCR was carried out in 13–14 cycles. Amplified DNA was purified with 1.2× Ampure beads and eluted into 12.5 µl of nuclease-free water. Purified DNA solutions from the eight aliquots were combined and loaded into five lanes of 4% E-gel (Invitrogen, G401004). DNA bands between 300 bp and 1.2 kb were excised. DNA was extracted using the NEB Monarch gel purification kit (NEB, T1020S) with two columns and eluted in 30 µl of elution buffer.

#### Sequencing

The molarity of the sequencing library was measured using a Qubit 4.0 Fluorometer (Invitrogen, Q33238) and Qubit dsDNA HS assay kit (Invitrogen, Q33231). Fragment size distribution was assessed using an Agilent bioanalyser with high-sensitivity DNA chromatin immunoprecipitation sequencing. The library was sequenced by UC San Diego IGM Genomics Center using an Illumina NovaSeq 6000. The sequencer was set to read a 28 bp sequence next to the universal adaptor as Read1, an 8 bp index sequence from the I7 region inside the index adaptor and a 150 bp sequence next to the index adaptor as Read2.

### Computational analysis

#### The MUSIC-docker data-processing pipeline

We developed MUSIC-docker to process MUSIC sequencing data using Docker to encapsulate a Snakemake^57^ pipeline, ensuring cross-platform execution. This handles I7 index-split, paired-end fastq files, processes them separately into RNA and DNA sequences, adds cell and complex barcodes and maps to the genome, removing PCR duplicates and deriving processed files. Detailed documentation is available at http://sysbiocomp.ucsd.edu/public/wenxingzhao/MUSIC_docker/intro.html and Supplementary Note 14.

The raw sequencing output (.bcl) was converted to FASTQ files with bcl2fastq, producing eight FASTQ files with 28 bp read1 and 150 bp read2. Read1 includes the 10X barcode and 10X UMI, with read2 containing cell barcodes, the RNA and DNA linkers and insert sequences.

The demultiplexing step extracts cell and complex barcode information and fragment identity information, creating separate FASTQ files for RNA and DNA reads individually in which the read sequence will be the insert and the read name will be the fragment identity information.

To address potential artificial sequences introduced by sequencing errors and experimental design, we remove consecutive As or Gs from the 3′ end of DNA inserts if their length is greater than 20 bp. For RNA reads we detect the ssDNA region of the RNA linker sequence CGAGGAGCGCTT and remove any sequence following it. We use cutadaptor (v.2.8)^58^ with the parameter ‘-q 15 -m 20’. DNA and RNA inserts are mapped to the genome using bowtie2 (v.5.4.0)^59^ with the parameters ‘bowtie2 -p 10 -t --phred33 -x’, and bwa (v.0.7.17)^60^ mem with parameters ‘-SP5M’, respectively. Uniquely mapped reads are selected for downstream analysis.

Following reads mapping, PCR duplicates, identified by shared 10X UMI and mapped coordinates, are removed using customized script. This script sorts the BAM file, scans it once and flags duplicates if they meet specific criteria: (1) it maps to a location within 8 bp of the previous read; (2) it shares the same cell and molecular barcodes with the previous read; and (3) its UMI exhibits a Levenshtein distance of less than 2 bp from the UMI of the previous read. All identified PCR duplicates are subsequently removed to ensure the integrity of downstream analyses.

Finally, deduplicated BAM files from each I7 index library are merged into a comprehensive, sorted BAM file capturing essential information for both DNA and RNA, including cell and molecular barcodes (10X and I7 index) and insert mapping location.

#### Mixed-species experiment

Based on a previously published method^12^, we assigned a cell to a species if 95% of its DNA reads could map to a single species. Cells with uniquely mapped, non-duplicated DNA reads of less than 1,000 were classified as ambient cells. To calculate single-cluster mixed-species rate we assigned a cluster to a single species if more than 99% of its uniquely mapped non-duplicated DNA reads came from that species.

#### Parsing multiplex clusters into pairwise interactions with normalization

Each cluster is a collection of reads that share the same cell, 10X and I7 barcodes (CB + GEM + I7). A cluster of two reads (cluster size 2) corresponds to a pairwise interaction and a cluster of three or more reads corresponds to a multiplex cluster. Each multiplex cluster is decomposed into pairwise interactions with a normalization procedure that adjusts for the total number of combinations of pairwise interactions from a multiplex cluster, as previously described^12,22^. For homotypical clusters, which are clusters containing exclusively DNA or RNA reads, each homotypical cluster of size *N* is first decomposed into all non-overlapping pairs and then each pair is normalized by a factor of 1/*N*. For heterotypic clusters, which are clusters containing both DNA and RNA reads, each heterotypic cluster is first decomposed into all DNA–RNA pairs and then each pair is normalized by a factor of 1/(*M* + *N*), where *M* and *N* are the numbers of DNA and RNA reads, respectively. This normalization process removes the difference in the number of pairwise decompositions from different-sized clusters, thus ensuring that larger clusters are not inflated in regard to the number of decomposed pairwise interactions^12,22^.

To generate a two-dimensional contact map of DNA–DNA interactions we first define the size of each unit, typically represented as genomic bins, for rows and columns. The weight assigned to bin [*i*, *j*] is determined by the total number of clusters containing DNA reads mapped to both the *i*th and *j*th bin, and this is normalized by cluster size as previously described. To compare DNA–DNA contact differences within a specific genomic region, cluster sizes are calculated using only DNA reads mapped to that region, considering small, medium and large clusters. If the DNA–DNA contact map is generated for multiple cells, the weighted sum of all clusters is calculated. Similar methods are applied to derive RNA–DNA two-dimensional contact maps, with the calculation of the weight of each interaction adjusted accordingly.

To determine RAL at specific genomic bins, representing one-dimensional RNA–chromatin contacts for a particular RNA of interest, we calculate the total weighted RNA–DNA interactions involving the RNA of interest and DNA ends mapped to the desired genomic bins. For ensemble maps, RAL values from individual cells are summed.

Finally, for visualization of the two-dimensional contact maps of DNA–DNA or RNA–DNA interactions, the raw contacts obtained following previously mentioned procedures are scaled up using a linear factor, typically 100 or 1,000. This amplification step prevents the application of logarithmic transformation to decimal values. Following amplification, a logarithmic transformation is performed to enhance visualization of the contact maps.

#### Comparison of MUSIC DNA–DNA contacts with Micro-C data

To achieve consistency in the scale of total contacts between MUSIC and Micro-C, contact maps obtained from both methods underwent a standardized transformation. Initially the contact maps derived from Micro-C or MUSIC were logarithmically scaled; these were then normalized to their respective maximum values, resulting in all contacts being constrained within the range of [0, 1]. The Micro-C data used in this study were obtained from the 4DN portal^61^ (4DNFI2TK7L2F)^34^. To extract raw contacts from the .hic file, the straw^62^ tool was employed.

To calculate the compartment score (PC1 score) for both Micro-C and MUSIC DNA–DNA contact matrices we first computed the expected contact matrix. Subsequently we determined the PC1 score from the correlation matrix of the observed/expected ratio matrices, in which the expected matrix was derived by calculating average contact frequencies as a function of genomic distances. For comparison of PC1 correlations between Micro-C and MUSIC, the calculations were performed individually on each chromosome. The reported correlation represents the median of these correlations across all chromosomes.

#### nsaRNA and pre-mRNA RAL

Pre-mRNAs are RNA reads that exhibit an overlap of at least 15 bp with gene introns and are classified as protein-coding RNAs. The calculation of RNA–DNA cluster weight involves the inclusion of all nsaRNAs and pre-mRNAs, along with their associated DNA reads. RNA–DNA clusters of size not exceeding 1,000 were selected for the computation of RAL. The H1 A/B compartment data used in this study were obtained from the 4DN data portal (4DNFID162B9J)^2^.

#### Calculation of genomic distance versus contact frequency curve from MUSIC data

The relationship between contact frequency and genomic distances within chromosomal arms was systematically examined. Initially the genomic distance range (from 10 bp to 150 megabases) was divided into 500,000 equally sized bins. Subsequently, for DNA reads originating from DNA–DNA or RNA–DNA clusters, the genomic distances of all intrachromosomal pairwise interactions were determined and the frequencies for each genomic distance bin were computed. These frequencies were then normalized based on the weight assigned to each interaction. Normalized frequencies were calculated for each genomic bin in every cluster and single cell. Finally, genomic distances versus contact frequencies were aggregated across all clusters from all single cells.

We used cooltools (v.0.5.4)^63^ to generate the curve in Micro-C data representing genomic distance versus contact frequency. We downloaded the .mcool file for H1 Micro-C from the 4DN data portal (4DNFI9GMP2J8)^34^.

#### Preprocessing and filtering of single brain cells

For each brain sample we applied standard MUSIC_docker pipelines to obtain valid RNA and DNA reads information for each single cell. To select high-quality single cells for robust analysis and interpretation of our data, we removed cells with fewer than 100 RNA reads or fewer than 5,000 DNA reads for downstream analysis on brain samples.

#### Transcriptome merging of brain samples

We first constructed the single-cell RNA expression count matrix by calculating the number of RNA reads mapped to each human gene (GENCODE^64^, v.36; chrM genes are excluded) for all single cells from all 14 brain samples. We then constructed one Seurat object (Seurat v.4.3.0)^65,66^ for each brain sample with the parameters ‘min.cells = 2 and min.features = 200’ (filtering out genes expressed in no more than two cells and filtering out cells with no more than 200 expressed genes). The count matrix from all brains was then integrated using RunHarmony from the harmony R package (v.0.1.1)^60^ based on STransform processed data, and regressed out on factors including individual library and experimental batches.

#### Expression of brain genes

For comparison of gene expression in brain cells we used the LogNormalize method from the Seurat R package. Raw reads counts were first normalized by library size and then log-transformed.

#### Single-cell clustering and cell type identification

The integrated brain object was then subjected to dimensionality reduction by UMAP methods based on the first 20 principal components from PCA using the Seurat R package. All cells are then clustered in an unsupervised method using a shared nearest-neighbour graph based on *k*-nearest neighbours (*k* = 20) calculated from the top two coordinates of UMAP. Clusters were then derived by optimization of the modularity function using the function FindClusters with parameter resolution at 0.05. Excitatory and inhibitory neurons were clustered by first extracting the subset of cells and reclustering at a resolution of 1.

We assigned cell types to each cluster by the known cell-type-specific marker gene expression level. Cell-type-specific marker genes are based on previous publications: for major brain cell types^38^, subclusters (Azimuth)^67^ and vascular cells^68^. Each cluster is assigned to one cell type (A) if two times the average expression of all marker genes of cell type A plus the proportion of cells in the cluster expressing marker genes for cell type A are higher than any other cell types. We designed this score that takes both cell type marker gene expression level and proportion of cells expressing the genes into consideration, but with a greater emphasis on expression level.

#### Single-cell LCS-erosion score and transcriptomic age calculation

We calculated the LCS-erosion score for each cell by determining the middle point of the genomic distances of the top ten most frequently contacting genomic bins. To derive the contact frequency versus genomic distances heatmap for all frontal cortex cells we first generated 149 genomic bins spanning from 5,000 bp to 150 megabase pairs, with the *n*th bin spanning the genomic region $({2}^{{\log }_{2}(5,000)+\frac{\{{{\rm{l}}{\rm{o}}{\rm{g}}}_{2}(1.5\times 1{0}^{8}\}-{{\rm{l}}{\rm{o}}{\rm{g}}}_{2}(5,000))}{150}\times (n-1)}$, ${2}^{{\log }_{2}(5,000)+\frac{\{{{\rm{l}}{\rm{o}}{\rm{g}}}_{2}(1.5\times 1{0}^{8}\}-{{\rm{l}}{\rm{o}}{\rm{g}}}_{2}(5,000))}{150}\times n})$, where *n* = 1,2,3,..,150. Subsequently, for DNA reads originating from DNA–DNA or RNA–DNA clusters, the genomic distances of all intrachromosomal pairwise interactions were determined and the frequencies for each genomic distance bin calculated. These frequencies were then normalized against bin size. For each single cell the total contact frequency was normalized against total frequency within each cell before plotting the heatmap. Cells exhibiting an LCS-erosion score exceeding 3 × 10^5^ were classified as LCS-eroded cells, with the remainder considered LCS-preserved.

For assessment of the transcriptomic-based biological age of cells we implemented the SCALE methodology described in a previous study^43^. This approach involves using a list of human ageing-associated genes obtained from https://sysomics.com/AgingMap/. Initially we determined the direction (either +1 or −1) of each marker gene based on its correlation with the chronological age of the sample. This direction depended on whether gene expression was positively (+1) or negatively (−1) correlated with age. Subsequently, for each marker gene, we assigned a weight computed as the proportion of cells expressing that gene multiplied by its directional value. Finally we calculated the transcriptomic age for each cell using the dot product of gene expression *z*-scores and corresponding gene weights.

#### LCS-erosion score-associated transcriptome functions

To identify genes whose expression is significantly associated with chromatin LCS-erosion score, we applied analysis of variance (ANOVA) between the expression of each gene and LCS-erosion score across all cells. Genes with *P* < 0.01, *F* > 1 and the absolute value of Spearman correlation between LCS-erosion score and gene expression greater than 0.1 were considered significant. To identify cell-type-specific LCS-erosion score-associated genes we applied ANOVA individually within each cell type. We used the R package gprofiler2 (ref. ^69^) for pathway enrichment analysis. Enriched wikipathway, KEGG and REACtom pathways are shown.

#### eQTL analysis

All brain cell-type-specific eQTL data were downloaded from ref. ^45^. We compiled a combined dataset by selecting eQTLs with nominal *P* < 1 × 10^−4^ and removing those in endothelial cells and pericytes. MUSIC DNA–DNA contacts between eQTL and their target genes are read pairs where one end is mapped (one base overlap) over the eQTL and the other over the gene promoter (2.5 kb flanking region from the transcriptional start site). The global chi-square test was performed on a 6 × 6 table to test the overall association of cell-type-specific DNA–DNA contacts with cell-type-specific eQTL–gene pairs. The 6 × 6 table was then reduced to six 2 × 2 tables to test that association in each cell type (chi-square test). The 95% confidence interval of the odds ratio was calculated as exp(log(odds ratio) ± 1.96 × SELOR), where SELOR is the standard error of log(odds ratio).

#### Analysis of XIST–chromatin interactions

To derive the one-dimensional XIST-genome RAL we extracted all clusters containing at least one XIST RNA. For each cluster with *M* XIST RNA reads and *N* DNA reads we derived *M* × *N* paired XIST–DNA interactions, each of which was then normalized according to its cluster size 1/(*M* + *N*). We then binned the whole genome at 1 Mb resolution. The XIST RAL for each bin is the total weight of all XIST–DNA interactions with DNA ends overlapped with that bin.

To derive the two-dimensional RNA–DNA contact map for chromosome X we extracted all RNA and DNA reads that can map to chromosome X. Next, for any matched molecular complex barcode with *M* chromosome X RNA reads and *N* chromosome X DNA reads, we again first derived all combinations of RNA–DNA interactions, with adjusted weight indicating the reverse of cluster size (1/*M* + *N*). We binned chromosome X at 1 Mb resolution; *M*[*i*, *j*] then represents the sum of weighted interactions whose RNA ends mapped to the *i*th bin and DNA ends mapped to the *j*th column.

#### XAL stratification and corresponding genomic distance versus contact frequency

XIST–chromosome X association levels are numerically represented as the number of XIST-attached chromosome X DNA bins; each individual cell will have one XAL value. To assess the differences in chromatin organization between XIST^+^ and XIST^−^ clusters, we stratified all brain cells into four groups based on their XAL value: group 1, with zero XAL, which includes all cells with no detectable XIST–chromosome X association; and groups 2–4, with increasing XAL values, which include all cells with an XIST–chromosome X association, split equally between these three groups. To derive genomic distances and contact frequency relationships we follow the methods introduced in ‘Calculation of genomic distance versus contact frequency curve from MUSIC data’.

### Human sample acquisition

The acquisition of postmortem brain tissue was conducted at Banner Sun Health Research Institute with Institutional Review Board approval (study 1132516, investigator T. Beach). Informed consent was obtained from all tissue donors.

### Reporting summary

Further information on research design is available in the Nature Portfolio Reporting Summary linked to this article.

## Online content

Any methods, additional references, Nature Portfolio reporting summaries, source data, extended data, supplementary information, acknowledgements, peer review information; details of author contributions and competing interests; and statements of data and code availability are available at 10.1038/s41586-024-07239-w.

## Supplementary information

Supplementary Information Supplementary Notes 1–14, Tables 1–6, Figs. 1–3 and references.

Reporting Summary

## Data Availability

All processed data, including those from cell lines and brain samples and raw sequencing data from cell lines, have been deposited in the Gene Expression Omnibus (GEO) under accession GSE253754. Raw sequencing data for brain samples have been deposited in the HuBMAP data portal (https://portal.hubmapconsortium.org/) and dbGap phs003568.v1.p1 with controlled access. Please follow the NIH Delegated Acquisition Certification instructions to request authorized access. We also downloaded the following public single-cell gene expression datasets: CITE-seq, GSE100866 (PBMC); SNARE-seq, GSE126074 (AdBrainCortex); PairedTag, GSE152020; and snRNA-seq, syn18485175. Micro-C data were downloaded from the 4DN data portal under session no. 4DNFI9GMP2J8.

## Code availability

The pipeline used to process the raw data is documented on our website: http://sysbiocomp.ucsd.edu/public/wenxingzhao/MUSIC_docker/intro.html. We developed a MUSIC-docker for raw data processing, the code for which can be accessed at https://github.com/Zhong-Lab-UCSD/MUSIC-docker (ref. ^70^). Additional analysis scripts associated with the paper can be accessed at https://github.com/Zhong-Lab-UCSD/MUSIC-tools (ref. ^71^) and made available on request.
