# Supplementary material for: Single-cell multiplex chromatin and RNA interactions in ageing human brain
Source: Nature. 2024 Mar 27;628(8008):648–56. doi: 10.1038/s41586-024-07239-w (PMC11023937; doi:10.1038/s41586-024-07239-w)
Supplement: Supplementary file 2 — Reporting Summary [file 41586_2024_7239_MOESM2_ESM.pdf]

nature portfolio

Corresponding author(s): Sheng Zhong

Last updated by author(s): Feb 18, 2024

Reporting Summary

Nature Portfolio wishes to improve the reproducibility of the work that we publish. This form provides structure for consistency and transparency in reporting. For further information on Nature Portfolio policies, see our Editorial Policies and the Editorial Policy Checklist.

Statistics

For all statistical analyses, confirm that the following items are present in the figure legend, table legend, main text, or Methods section.

n/a	Confirmed	
☐	☒	The exact sample size (*n*) for each experimental group/condition, given as a discrete number and unit of measurement
☐	☒	A statement on whether measurements were taken from distinct samples or whether the same sample was measured repeatedly
☐	☒	The statistical test(s) used AND whether they are one- or two-sided<br>*Only common tests should be described solely by name; describe more complex techniques in the Methods section.*
☒	☐	A description of all covariates tested
☒	☐	A description of any assumptions or corrections, such as tests of normality and adjustment for multiple comparisons
☐	☒	A full description of the statistical parameters including central tendency (e.g. means) or other basic estimates (e.g. regression coefficient) AND variation (e.g. standard deviation) or associated estimates of uncertainty (e.g. confidence intervals)
☐	☒	For null hypothesis testing, the test statistic (e.g. *F*, *t*, *r*) with confidence intervals, effect sizes, degrees of freedom and *P* value noted<br>*Give P values as exact values whenever suitable.*
☒	☐	For Bayesian analysis, information on the choice of priors and Markov chain Monte Carlo settings
☒	☐	For hierarchical and complex designs, identification of the appropriate level for tests and full reporting of outcomes
☐	☒	Estimates of effect sizes (e.g. Cohen's *d*, Pearson's *r*), indicating how they were calculated

Our web collection on statistics for biologists contains articles on many of the points above.

Software and code

Policy information about availability of computer code

Data collection	The library was sequenced by UC San Diego IGM Genomics Center utilizing an Illumina NovaSeq 6000. The sequencer is set to read a 28 bp sequence next to the Universal Adapter as the Read1, a 8 bp index sequence from the I7 region inside the Index Adapter, and a 150 bp sequence next to the Index Adapter as the Read2.
Data analysis	Pipeline used to process the raw data is documented in our website: http://sysbiocomp.ucsd.edu/public/wenxingzhao/MUSIC_docker/intro.html. We developed a MUSIC-docker for the raw data processing and the code can be accessed at: https://github.com/Zhong-Lab-UCSD/MUSIC-docker. Additional analysis scripts associated with the paper can be accessed at: https://github.com/Zhong-Lab-UCSD/MUSIC-tools and made available upon request.<br><br>A customized pipeline called MUSIC-docker (http://sysbiocomp.ucsd.edu/public/wenxingzhao/MUSIC_docker/intro.html) is used to process the raw sequencing data to molecular identity resolved bam file. Within MUSIC docker, In house scripts were used to parse the raw fastq files and separate RNA and DNA inserts into separate fastq files. Cutadapt (2.8) is used to remove potential artifacts. For reads mapping, bowtie2 (5.4.0) is used for DNA inserts genome mapping with the command “bowtie2 -p 10 -t --phred33 -x ” and bwa mem (0.7.17) is used to map RNA inserts to the reference genome in a splice aware manner. Customized code is then used to remove PCR duplicates from mapping results. MUSIC-docker image can be downloaded from Docker Hub through "docker pull irenexzwen/MUSIC-docker".<br><br>Data were analyzed using customized R (4.1.0) scripts, exploiting several R packages including: GenomicRanges (1.38.0), chromstaR (1.20.2), plyranges (1.14.0), InteractionSet (1.14.0) as the main packages for genomic ranges data manipulation; KaryoploteR (1.12.4), ggplot2 (3.4.2), plotgardener (1.0.17) and ggpubr (0.6.0) for visualization of genomic data and tracks, ComplexHeatmap (2.10.0) to make heatmaps. gprofiler2 (0.2.2) for enriched pathway analysis. To analyze micro-C data, we used strawr (0.0.91) to extract the contact matrix from .hic file which is downloaded from 4DN data portal. Juicer Tools (1.22.01) was used for calling A/B compartment and loops from micro-c .hic file. A/B

compartments were called by Juicer's "Eigenvector" tool and loops were called by Juicer's "CPU HiCCUPS" tool, default parameters were used. We used cooltools (0.5.4) for generating the genomic distance versus contact frequency curve in Micro-C data. For single cell RNA expression analysis we used Seurat (4.3.0) for data storage, quality control, clustering and plot. We use harmony (0.1.1) for data integration.

For manuscripts utilizing custom algorithms or software that are central to the research but not yet described in published literature, software must be made available to editors and reviewers. We strongly encourage code deposition in a community repository (e.g. GitHub). See the Nature Portfolio guidelines for submitting code & software for further information.

Data

Policy information about availability of data

All manuscripts must include a data availability statement. This statement should provide the following information, where applicable:

- Accession codes, unique identifiers, or web links for publicly available datasets
- A description of any restrictions on data availability
- For clinical datasets or third party data, please ensure that the statement adheres to our policy

All processed data, including data from cell lines and brain samples, has been deposited in GEO (GSE253754). The raw sequencing data from cell lines has been deposited in GEO (GSE253754). Raw sequencing data for brain samples has been deposited in the HuBMAP data portal (https://portal.hubmapconsortium.org/) with controlled access. Please follow the NIH Delegated Acquisition Certification (DAC) instructions to request authorized access. We also downloaded public single cell gene expression datasets. CITE-seq: GSE100866(PBMC); SNARE-seq: GSE126074(AdBrainCortex); Paired-Tag: GSE152020; snRNA-seq: syn18485175. Micro-C data is downloaded from the 4DN data portal under session number: 4DNFI9GMP2J8.

Research involving human participants, their data, or biological material

Policy information about studies with human participants or human data. See also policy information about sex, gender (identity/presentation), and sexual orientation and race, ethnicity and racism.

Reporting on sex and gender	The acquisition of postmortem brain is conducted at Banner Sun Health Research Institute, under IRB approval for investigator Thomas Beach, MD, PhD. Snap frozen cortex tissues from age matched 7 females and 7 males were used to generate MUSIC library for each individual. In our preliminary submission, we examined the distribution of female and male cells within each brain cell type. We conducted a comparison of XIST expression levels between males and females. Subsequently, for the XIST RNA localization analysis, only female cells were included.
Reporting on race, ethnicity, or other socially relevant groupings	The metadata for the brain samples can be found in Supplementary Table 4, which is included in our initial submission. As per the sample demographics provided by the Banner Sun Health Research Institute, all individuals in the study are categorized as race 1. Race is not considered as a variable in our analysis.
Population characteristics	The comprehensive metadata for the brain samples can be found in Supplementary Table 4, which is included in our initial submission. Each individual's data includes age at death, ApoE genotype, Plaque Total level, Tangle Total level, and Braak score. The deceased age of the 7 female donors ranges from 59 to 82 years, with one donor being 90 years or older. Similarly, the deceased age of the 7 male donors ranges from 63 to 81 years, with one donor being 90 years or older. The ApoE genotype distribution among the female donors includes 2/3, 3/3, 3/3, 3/3, 3/3, 3/3, and 3/4, while among the male donors it includes 2/3, 2/4, 3/3, 3/3, 3/3, 3/4, and 4/4.<br><br>Plaque Total represents the cumulative score obtained by summing the scores from Plaque F, T, P, H, and E, which are used to assess senile plaque density in standard regions of the frontal, temporal, and parietal lobes, as well as the hippocampal CA1 region and entorhinal/transentorhinal region. Tangle Total, similarly, is derived by summing the scores from Tangle F, T, P, H, and E. The Braak score corresponds to the neurofibrillary stage (ranging from 0 to VI) as originally defined by Braak and Braak (1991). This score is obtained using thick 40-80 micron sections stained with Gallyas, Campbell-Switzer, and thioflavine S stains.<br><br>It is important to note that our analysis does not differentiate patients based on their disease states.
Recruitment	The recruitment process for the BBDP subjects primarily involves the enrollment of cognitively normal volunteers residing in retirement communities within metropolitan Phoenix, Arizona. Additionally, specific recruitment efforts are targeted towards individuals diagnosed with Alzheimer's disease, Parkinson's disease, and cancer. The median age at death for the enrolled subjects is 82 years. Throughout their lives, subjects in the study undergo standardized assessments encompassing general medical, neurological, neuropsychological, and movement disorders evaluations. After death, more than 90% of the participants receive comprehensive pathological examinations conducted by pathologists who hold valid medical licenses. Certain subsets of the Program are utilized by esteemed institutions such as the US National Institute on Aging Arizona Alzheimer's Disease Core Center and the US National Institute of Neurological Disorders and Stroke National Brain and Tissue Resource for Parkinson's Disease and Related Disorders.
Ethics oversight	The acquisition of postmortem brain is conducted at Banner Sun Health Research Institute with IRB approval (Study Number: 1132516, Investigator: Thomas Beach, M.D., Ph.D.). Informed consent was obtained from all tissue donors.

Note that full information on the approval of the study protocol must also be provided in the manuscript.

Field-specific reporting

Please select the one below that is the best fit for your research. If you are not sure, read the appropriate sections before making your selection.

☒ Life sciences ☐ Behavioural & social sciences ☐ Ecological, evolutionary & environmental sciences

For a reference copy of the document with all sections, see nature.com/documents/nr-reporting-summary-flat.pdf

Life sciences study design

All studies must disclose on these points even when the disclosure is negative.

Sample size	Fourteen human frontal cortex samples. No sample size calculation was performed. The number of samples was bounded by our limited access to brain specimens. We aimed to characterize more than 1,000 single cells. The data resolved more than 9,000 single cells and thus satisfied our goal.
Data exclusions	No data were excluded.
Replication	MUSIC is applied to analyze 14 human frontal cortex samples.
Randomization	Randomization is not applicable because this study does not involve any treatment or modifiable environmental factors.
Blinding	Blinding is not applicable because this study does not involve any treatment or modifiable environmental factors.

Reporting for specific materials, systems and methods

We require information from authors about some types of materials, experimental systems and methods used in many studies. Here, indicate whether each material, system or method listed is relevant to your study. If you are not sure if a list item applies to your research, read the appropriate section before selecting a response.

Materials & experimental systems

n/a	Involved in the study	
☒	☐	Antibodies
☐	☒	Eukaryotic cell lines
☒	☐	Palaeontology and archaeology
☒	☐	Animals and other organisms
☒	☐	Clinical data
☒	☐	Dual use research of concern
☒	☐	Plants

Methods

n/a	Involved in the study	
☒	☐	ChIP-seq
☒	☐	Flow cytometry
☒	☐	MRI-based neuroimaging

Eukaryotic cell lines

Policy information about cell lines and Sex and Gender in Research

Cell line source(s)	H1 human embryonic stem cells were obtained from the 4D Nucleome Consortium. E14 mouse embryonic stem cells were obtained from Bing Ren lab.
Authentication	Cell lines are authenticated by morphology. Cell passage number was kept under P10. Additionally, karyotype evidence is derived from the genomics data of this work.
Mycoplasma contamination	Our lab routinely tests for mycoplasma contamination on all cultured cells on a quarterly basis.
Commonly misidentified lines (See ICLAC register)	H1 and E14 cell lines are not commonly misidentified lines.
